# Molecular Evidence for the Inverse Comorbidity between Central Nervous System Disorders and Cancers Detected by Transcriptomic Meta-analyses

**DOI:** 10.1371/journal.pgen.1004173

**Published:** 2014-02-20

**Authors:** Kristina Ibáñez, César Boullosa, Rafael Tabarés-Seisdedos, Anaïs Baudot, Alfonso Valencia

**Affiliations:** 1 Structural Biology and Biocomputing Programme, Spanish National Cancer, Research Centre (CNIO), Madrid, Spain; 2 Department of Medicine, University of Valencia, CIBERSAM, INCLIVA, Valencia, Spain; 3 Aix-Marseille Université, CNRS, I2M, UMR 7373, Marseille, France; University of Washington, United States of America

## Abstract

There is epidemiological evidence that patients with certain Central Nervous System (CNS) disorders have a lower than expected probability of developing some types of Cancer. We tested here the hypothesis that this *inverse comorbidity* is driven by molecular processes common to CNS disorders and Cancers, and that are deregulated in opposite directions. We conducted transcriptomic meta-analyses of three CNS disorders (Alzheimer's disease, Parkinson's disease and Schizophrenia) and three Cancer types (Lung, Prostate, Colorectal) previously described with *inverse comorbidities*. A significant overlap was observed between the genes upregulated in CNS disorders and downregulated in Cancers, as well as between the genes downregulated in CNS disorders and upregulated in Cancers. We also observed expression deregulations in opposite directions at the level of pathways. Our analysis points to specific genes and pathways, the upregulation of which could increase the incidence of CNS disorders and simultaneously lower the risk of developing Cancer, while the downregulation of another set of genes and pathways could contribute to a decrease in the incidence of CNS disorders while increasing the Cancer risk. These results reinforce the previously proposed involvement of the *PIN1* gene, Wnt and P53 pathways, and reveal potential new candidates, in particular related with protein degradation processes.

## Author Summary

A lower-than-expected probability of developing certain types of Cancer has been observed in patients with CNS disorders, including Alzheimer's disease, Parkinson's disease or Schizophrenia. Understanding such a protective effect could be the key to finding novel treatments for both types of conditions, for instance thanks to drug repurposing. However, little is known about the underlying mechanisms for these intriguing *inverse comorbidities*. Although environmental causes, drug treatments or lower screening surveys might contribute to the *inverse comorbidity* between complex disorders, we propose that *inverse comorbidity* is, at least in part, due to genetic factors.

We observe here that a common set of genes and biological processes are deregulated in opposite directions in CNS disorders and Cancers, *i.e.* upregulated in CNS disorders and downregulated in Cancers, or vice versa. We propose the alluring hypothesis that the deregulation of these genes and processes could promote CNS disorders and simultaneously lower the initiation or progression of Cancers.

## Introduction

Epidemiological evidences point to a lower-than-expected probability of developing some types of Cancer in certain CNS disorders, including Alzheimer's disease (AD), Parkinson's disease (PD) and Schizophrenia (SCZ) [1]–[6]. Our current understanding of such *inverse comorbidities* suggests that this phenomenon is influenced by environmental factors, drug treatments and other aspects related with disease diagnosis. Genetics can additionally contribute to the *inverse comorbidity* between complex diseases, together with these external factors (for review, see [3]–[7]). In particular, we propose the deregulation in opposite directions of a common set of genes and pathways as an underlying cause of *inverse comorbidities*.

To investigate the biological plausibility of this hypothesis, a basic initial step is to establish the existence of inverse gene expression deregulations (*i.e.*, down- versus up-regulations) in CNS disorders and Cancers. Towards this objective, we have performed integrative meta-analyses of collections of gene expression data, publically available for AD, PD and SCZ, and Lung (LC), Colorectal (CRC) and Prostate (PC) Cancers. Clinical and epidemiological data previously reported *inverse comorbidities* for these complex disorders, according to population studies assessing the Cancer risks among patients with CNS disorders [8]–[17].

## Results and Discussion

**Figure 1:**
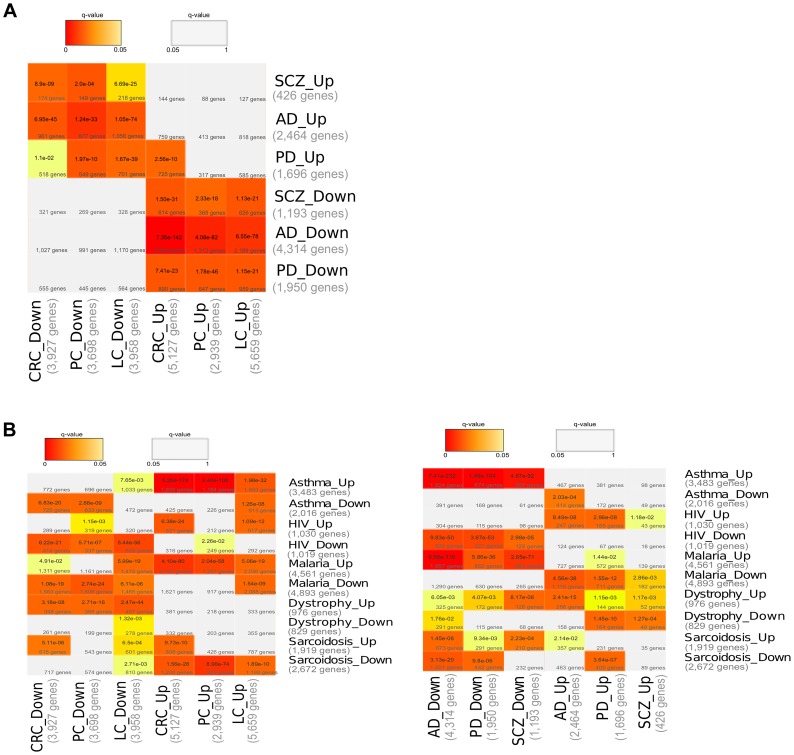
Comparisons of Differentially Expressed Genes (DEGs). (**A**) Comparisons of DEGs associated with Central Nervous System (CNS) disorders and Cancers. The DEGs identified as significantly up- and down-regulated (q-value<0.05) after gene expression meta-analysis in each CNS disorder (Alzheimer's Disease, AD; Parkinson's Disease, PD; and Schizophrenia, SCZ) and Cancer type (Lung Cancer, LC; Colorectal Cancer, CRC; and Prostate Cancer, PC) are compared to each others. (**B**) Comparisons of DEGs between CNS disorders, Cancers and Asthma, HIV, Malaria, Dystrophy, Sarcoidosis. The DEGs identified as significantly up- and down-regulated (q-value<0.05) after gene expression meta-analysis in each CNS disorder (Alzheimer's Disease, AD; Parkinson's Disease, PD; and Schizophrenia, SCZ), Cancer type (Lung Cancer, LC; Colorectal Cancer, CRC; and Prostate Cancer, PC), and in Asthma, HIV, Malaria, Dystrophia and Sarcoidosis, are compared to each others. Cells are coloured according to the significance of the overlaps (Fisher's exact test, Bonferroni correction for multiple testing, see **Methods**). Grey cells correspond to non-significant overlaps (q-value>0.05).

For each CNS disorder and Cancer type independently, we undertook meta-analyses from a large collection of microarray gene expression datasets to identify the genes that are significantly up- and down-regulated in disease when compared with their corresponding healthy control samples (Differentially Expressed Genes – DEGs –, FDR corrected p-value (q-value)<0.05, see **Methods** and **Table S1**). Then, the DEGs of the CNS disorders and Cancer types were compared to each others. There were significant overlaps (Fisher's exact test, corrected p-value (q-value)<0.05, see **Methods**) between the DEGs upregulated in CNS disorders and those downregulated in Cancers. Similarly, DEGs downregulated in CNS disorders overlapped significantly with DEGs upregulated in Cancers (**Figure 1A**). Significant overlaps between DEGs deregulated in opposite directions in CNS disorders and Cancers are still observed while setting more stringent cutoffs for the detection of DEGs (qvalues lower than 0.005, 0.0005, 0.00005 and 0.000005, ****). A significant overlap between DEGs deregulated in the same direction was only identified in the case of CRC and PD upregulated genes (**Figure 1A**).

A molecular interpretation of the *inverse comorbidity* between CNS disorders and Cancers could be that the downregulation of certain genes would at the same time increase the risk of developing CNS disorders, while reducing the risk of developing Cancers. The upregulation of other genes would reduce the risk of developing CNS disorders and increase the risk of developing Cancers.

We then compared the CNS disorder and Cancer DEGs with DEGs of a number of diseases for which, to our knowledge, *inverse comorbidities* have not been reported in the literature. These diseases, for which large enough expression datasets were available, included Asthma, HIV, Malaria, Dystrophy and Sarcoidosis (see **Methods**). Significant overlaps were observed between DEGs of all these diseases and DEGS of CNS disorders or Cancers (**Figure 1B**). However, patterns of expression deregulation in opposite directions, which were found to be characteristic of the relation between CNS disorders and Cancers, are in most cases not observed with these other genetic or infectious diseases (**Figure 1B**). Indeed, the overlaps are predominantly significant between DEGs deregulated in the same directions, *i.e.* between upregulated genes of the different diseases (or conversely between down-regulated genes), and could be a signature of putative *positive comorbidities*. It is to note that Malaria and CNS disorders DEGs present overlaps between DEGs deregulated in opposite directions, contrarily to what is detected for other diseases. This observation will require additional research.

Overall, these observations support the indication of a signature for *inverse comorbidity* in gene expression deregulations in opposite directions.

The *PIN1* gene has been proposed previously as a putative link between the pathogeneses of AD and Cancer [4]. Through the isomerization of a proline preceded by phosphorylated Ser/Thr residues, the PIN1 protein is known to be a key regulator of cell division [18]. *PIN1* gene is typically overexpressed in human Cancers and as such, it has been assessed as a potential target for anticancer drugs [4]. In addition, PIN1 is depleted in AD, it has been shown to restore the function of the phosphorylated tau protein, and mouse models in which this protein is knocked-down present neurodegenerative phenotypes [18]–[19]. Our transcriptomic meta-analyses confirm and extend these observations as the expression of *PIN1* is downregulated in AD and PD, and upregulated in CRC (**Table S2**). Another interesting case is the *ATP13A2* gene, involved in the intracellular cation homeostasis. *ATP13A2* is part of a list established by Devine et al. of familial PD genes frequently mutated in Cancers [5]. Indeed, loss-of-function mutations of *ATP13A2* have been associated with early-onset Parkinsonism, and somatic mutations have been independently observed in Cancer [5]. We identified *ATP13A2* as downregulated in AD and PD, and upregulated in the three Cancer types considered (**Table S2**).

In the light of these findings, our approach appears to be capable of identifying candidate genes potentially associated with *inverse comorbidity*. In particular, 74 genes may be of interest since they are simultaneously downregulated in the three CNS disorders and upregulated in the three Cancer types examined (**Table 1**). RNA splicing (four genes: *PPIH*, *LSM4*, *NUDT21*, *SRSF2*) and aminoacyl t-RNA ligases (three genes: *FARSA*, *IARS*, *IARS2*) represent particularly interesting functions.

**Table 1:** DEGs significantly downregulated in the three CNS disorders and upregulated in the three Cancer types (q-value<0.05).

PPIAP11, IARS, GGCT, NME2, GAPDHP1, CDC123, PSMD8, MRPS33, FIBP, OAZ2, IARS2, SLC35B1, APOO, TMEM189-UBE2V1, VDAC1, TMED3, SMS, DNM1L, PRPS1, SRSF2, TMEM14D, TOMM70A, ATP6V1C1, NUP93, MRPL15, UBA5, PPIH, SMYD3, NIT2, SRD5A1, NUDT21, MRPL12, EEF1E1, MRPS7, TTPAL, BZW1P2, RP11-552M11.4, TSN, MECR, ZWINT, RPRD1A, UCHL5, NHP2P2, TFB2M, FEN1, CGREF1, IMPAD1, ARL1, ACLY, MRPL42, LSM4, KPNA1, TIMM23B, RP11-164O23.5, RP11-762H8.2, FARSA, MRPL4, API5, RP3-425P12.4, RFC3, RANBP9, TFCP2, GMDS, CCNB1, TMEM177, GUF1, HSPA13, NMD3, GCFC2, TUBGCP5, TBCE, YKT6, PHF14, BRCC3

We also pinpoint two genes involved in lipid biogenesis (*ACLY* and *MECR*), and other two are transcription factors: *NME2* and *TFCP2*, for which a genetic association with AD is debated [20]. Finally, two other genes, *OAZ2* and the spermine synthase *SMS*, are dedicated to polyamine metabolic processes. Interestingly, defects in the spermine synthase gene are associated with the X-linked mental retardation Snyder-Robison syndrom [21], and spermine is often the most abundant polyamine in Cancers [22]. The polyamine metabolic process hence may play a role in the pathological mechanisms of both CNS disorders and Cancers.

Conversely, 19 genes are simultaneously upregulated in the three CNS disorders and downregulated in the three Cancer types examined (**Table 2**), including for instance six genes involved in signal transduction (*TNFRSF1A*, *CDKN1A*, *NFKBIA*, *PTH1R*, *IL4R*, *MID1*). Particularly, *NFKBIA* is an interesting candidate because this gene is often deleted in glioblastoma [23], although to our knowledge no mutations or polymorphisms have been described in CNS disorders.

**Table 2:** DEGs significantly upregulated in the three CNS disorders and downregulated in the three Cancer types (q-value<0.05).

MT2A, MT1X, NFKBIA, AC009469.1, DHRS3, CDKN1A, TNFRSF1A, CRYBG3, IL4R, MT1M, FAM107A, ITPKC, MID1, IL11RA, AHNAK, KAT2B, BCL2, PTH1R, NFASC

In order to enhance the functional interpretation of the molecular bases of *inverse comorbidity*, we broaden the comparisons of expression deregulations by considering pathways instead of individual genes [24].

**Figure 2:**
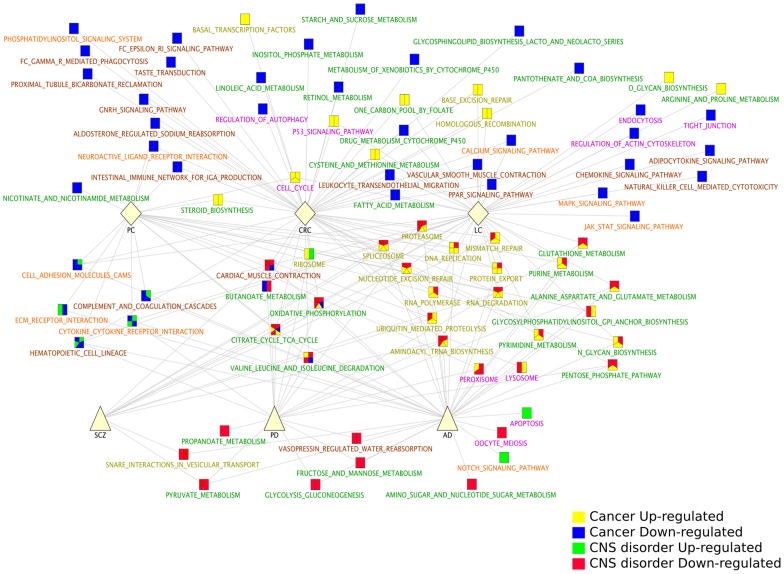
KEGG pathways significantly deregulated in Central Nervous System (CNS) disorders and Cancer types. KEGG pathways [24] significantly up- and downregulated in each disease were identified using the GSEA method [34] (q-value<0.05). The significant pathways were compared between the 6 diseases and combined in a network representation. Node pie charts are coloured according to the pathway status as Cancer upregulated (yellow), Cancer downregulated (blue), CNS disorder upregulated (green) and CNS disorder downregulated (red). The green/blue and yellow/red associations thus correspond to pathways deregulated in opposite directions in CNS disorders and Cancers. Pathway labels are coloured according to their classifications provided by KEGG [24], as: Metabolism (green), Genetic Information Processing (yellow), Cellular Process (pink), Environmental Information Processing (red) and Organismal Systems (dark red). All networks are available at bioinfo.cnio.es/people/cboullosa/validation/cytoscape/Ibanezetal.zip, in cytoscape format (http://www.cytoscape.org/).

We identified the pathways that were significantly up- and downregulated (GSEA analyses, q-value<0.05, see **Methods** and **Table S3**) in each of the six diseases independently. Among all the KEGG [25] pathways significantly up- and down-regulated in the 6 diseases, 30 are shared by CNS disorders and Cancers (*i.e.*, significantly deregulated in at least 1 CNS disorder and 1 Cancer type). Strikingly, of these 30 shared pathways, 24 (80%) are deregulated in opposite directions in CNS disorders and Cancers (**Figure 2**, 63% and 86% for the Biocarta (http://www.biocarta.com/) and Reactome [26] databases, respectively, **Figure S2**).

The p53 signalling pathway is an anticipated candidate for deregulations in these diseases and for a role in *inverse comorbidity* [4]. Indeed, deregulations of the p53 signalling pathway are associated with the initiation and progression of Cancers, while recent studies also point to a role for this pathway in CNS disorders [27]. As such, specific polymorphisms in the *TP53* gene are found in SCZ patients [27]. Although the *TP53* gene itself does not appear to be differentially regulated in our analysis, the p53 pathway is upregulated in CRC and LC, while it is downregulated in PD, AD and SCZ (Reactome database; **Figure S2**, **Table S3**).

Similarly, the Wnt pathway may be particularly relevant as mutations in the genes encoding APC and β-catenin, elements of the Wnt pathway, have been described in CRC, while β-amyloid induced neurotoxicity in AD has been associated with impaired Wnt signalling [4], [18]. Furthermore, alterations in the Wnt signalling pathway are known to be involved in SCZ [28]. In our meta-analyses, we found the Wnt pathway to be downregulated in AD and PD, and upregulated in CRC (Reactome database; **Figure S2**).

Aside the Wnt and p53 pathways, our analysis reveals other pathways related to protein folding and protein degradation displaying patterns of downregulation in CNS disorders and upregulation in Cancers, and that may be relevant for *inverse comorbidity*. For instance, the Ubiquitin/Proteasome system is consistently downregulated in CNS disorders and upregulated in Cancers according to the three pathway databases analyzed (**Figure 2**, **Figure S2**, **Table S3**). The inverse relationship between the levels of expression deregulations of these pathways possibly suggests opposite roles in CNS disorders and Cancers.

**Figure 3:**
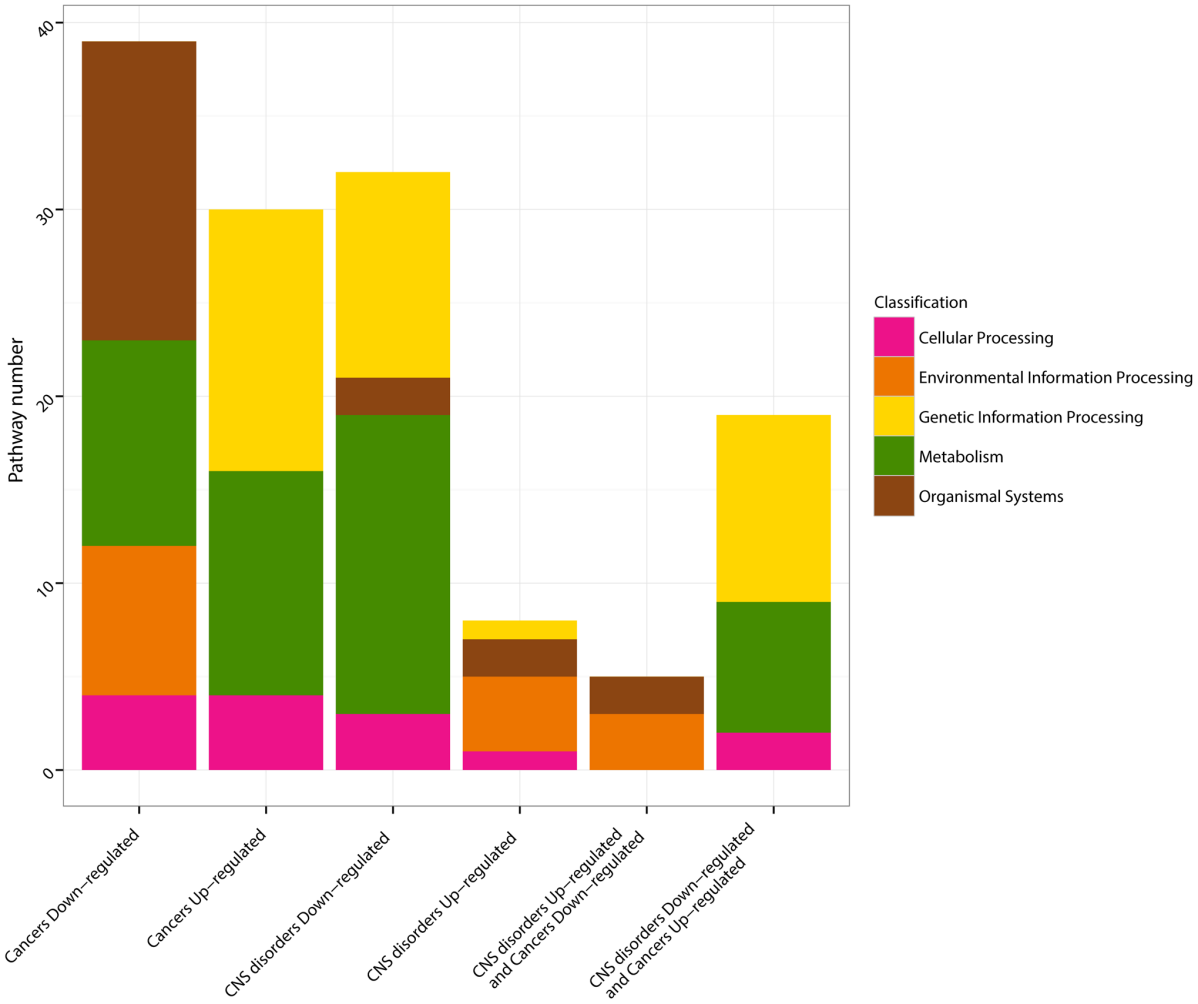
KEGG pathway classifications. The KEGG pathways [24] identified by the GSEA analysis [34] as significantly up- and down-regulated in CNS disorders, in Cancers, and simultaneously up-regulated in CNS disorders/down-regulated in Cancers, and down-regulated in CNS disorders/up-regulated in Cancers (q-values<0.05, **Figure 2**) are classified as Metabolism (green), Genetic Information Processing (yellow), Cellular Process (pink), Environmental Information Processing (orange) and Organismal Systems (dark red), according to the classification provided by KEGG.

A detailed examination of the KEGG pathways deregulated in opposite directions in CNS disorders and Cancers finally revealed that 89% of the KEGG pathways that were upregulated in Cancers and downregulated in CNS disorders are related to Metabolism and Genetic Information Processing (**Figure 2**, **Figure 3**). By contrast, the pathways downregulated in Cancers and upregulated in CNS disorders are related to the cell's communication with its environment (Environmental Information Processing and Organismal System; **Figure 2**, **Figure 3**). Hence, global regulations of cellular activity may account for a protective effect between inversely comorbid diseases.

Further analyses will be necessary to conclude to a direct protective effect of gene expression deregulations in cancer-prone tissues of patients suffering from CNS disorders. Indeed, the DEGs analyzed in this study are computed for each disease in the corresponding affected tissues, and cannot be extrapolated to gene expression deregulations in other tissues of the same patients. However, despite these limitations, the identification of antagonistically deregulated genes and pathways in complex diseases that have been previously described as *inversely comorbid* provides, to our knowledge, the first systematic insights into the possible molecular basis of these associations.

It suggests that the upregulation of a set of genes or processes could increase the incidence of CNS disorders and simultaneously lower the chances of developing Cancers, while the downregulation of another set of genes or processes could contribute to a decrease in the incidence of CNS disorders while increasing the Cancer risks.

The individuals delivering post-mortem brain samples in CNS disorders, or tumor tissues in the case of Cancers, are likely to have received drug treatments. Hence, the observed expression deregulations could be the consequence of the drugs administered to the patients. If this is the case, it can be hypothesized that some of the drugs used to treat CNS disorders might be able to revert the expression of a number of Cancer genes. In this context, the repurposing of drugs from the CNS to the Cancer field could open new therapeutic avenues. Indeed some punctual observations have been made. For example, the thioridazine, an anti-psychotic drug antagonizing the dopamine receptor and potentially able to alter physiological states and expression patterns, have been reported to target cancer stem cells selectively [29].

Finally, the analyses of inverse expression deregulations could serve as a new approach to investigate possible relations between complex diseases, of which the ones reported here between CNS disorders and Cancers can be considered as an initial example.

## Methods

The analysis pipeline (**Text S1**, workflow) is available as literate programming file describing the different steps of the analysis, together with the dynamic report (**Text S2**, knitr (http://yihui.name/knitr/) that can be used directly in R.)

### Gene expression data

Gene expression raw data (CEL files) were downloaded from NCBI GEO omnibus (GEO, http://www.ncbi.nlm.nih.gov/geo/), EBI ArrayExpress (AE, http://www.ebi.ac.uk/arrayexpress/) and Stanley Medical Research Institute, Online Genomics Database (SMRI, https://www.stanleygenomics.org) for Colorectal (CRC), Lung (LC) and Prostate (PC) Cancers, Alzheimer's disease (AD), Parkinson's disease (PD) and Schizophrenia (SZC), and for Asthma, HIV, Malaria, Dystrophy, Sarcoidosis (**Text S1**). For each disease, studies were filtered to select only the ones profiling at least 9 samples for disease and control cases, with Affymetrix arrays (GeneChip Human Genome U133 Plus 2.0, GeneChip Human Genome U133A and GeneChip Human Genome U133A 2.0 containing 23,945, 14,538 and 14,538 genes, respectively). For CNS disorders, only studies that measure gene expression in brain tissues were selected. For Cancers, only gene expression studies carried out in the LC, CRC and PC tumor tissues were considered.

### Microarray gene expression preprocessing and meta-analyses

The collected microarray data from the different studies were normalized with frozen Robust Multiarray Analysis (fRMA) [30] from the R Affy package [31]. Then, microarray meta-analyses were undertaken for each disease independently using the R MetaDE package [32]. MetaDE implements meta-analysis methods for differential expression analysis, and we used the Fixed Effects Model (FEM) [33]. This model assumes that the standardized effect sizes can be combined between the different studies, and that the variations in observed effects are only due to random error [34]–[35].

Similar results were obtained with the Random Effects Model (REM) approach that allows heterogeneity in the effect sizes between the different datasets (unpublished observations).

The meta-analyses led to the identification of genes up- and down-regulated in each disease, and significant differentially expressed genes (DEGs) were selected as those displaying a FDR corrected p-value (q-value)<0.05. Four other q-value cutoffs (0.005, 0.0005, 0.00005 and 0.000005) were selected to validate our results on more stringent DEGs sets (**Figure S1**).

### Comparisons of DEGs between the different diseases

Each CNS disorder DEGs were compared to each Cancer type DEGs, and the significances of the overlaps between the DEGs were assessed by a one-tailed Fisher's exact test, corrected for multiple testing by the Bonferroni approach (**Figure 1A** **, Figure S1**). The background number of genes necessary for the Fisher's test was set to 14,538.

The same procedure was applied for Cancers, CNS disorders and Asthma, HIV, Malaria, Dystrophy and Sarcoidosis (**Figure 1B**).

### GSEA analyses

For each CNS disorder and Cancer type independently, a gene set enrichment analysis was undertaken using GSEA [36] on the output of the meta-analyses, and focusing on KEGG [24], Biocarta (http://www.biocarta.com/) and Reactome [26] pathway databases. Significant pathways were selected as those with q-value (FDR)<0.05. Significant pathways in each disease were then compared to each others, and a network of pathways was built (**Figure 2** **, Figure S2**).

For the KEGG pathways, further classification of the pathways in Metabolism, Genetic Information Processing, Cellular Processes, Environmental Processes and Organismal Processes, as provided by KEGG, was done (**Figure 2**, **Table S2**). Pathways corresponding to Human Diseases were discarded.

## Supporting Information

Figure S1 Comparisons of Differentially Expressed Genes (DEGs) associated with Central Nervous System (CNS) disorders and Cancers at different q-value thresholds. The DEGs up- and down-regulated after gene expression meta-analysis in each CNS disorder (Alzheimer's Disease, AD; Parkinson's Disease, PD; and Schizophrenia, SCZ) and in each Cancer (Colorectal Cancer, CRC; Prostate Cancer, PC; Lung Cancer, LC) are selected for the 0.005 (a), 0.0005 (b), 0.00005 (c) and 0.000005 (d) thresholds, and compared to each others.
(PDF)

Figure S2 Biocarta and Reactome pathways significantly deregulated in the three types of Cancers and CNS disorders. Biocarta pathways (http://www.biocarta.com/) and Reactome pathways [26] Cancer upregulated (yellow), Cancer downregulated (blue), CNS disorder upregulated (green) and CNS disorder downregulated (red). The green/blue and yellow/red associations thus correspond to pathways deregulated in opposite directions in CNS disorders and Cancers. All networks are available at bioinfo.cnio.es/people/cboullosa/validation/cytoscape/Ibanezetal.zip, in cytoscape format (http://www.cytoscape.org/).
(PDF)

Table S1 Differentially Expressed Genes (DEGs) in Alzheimer's disease (AD), Parkinson's disease (PD), Schizophrenia (SCZ), Lung Cancer (LC), Prostate Cancer (PC), Colorectal Cancer (CRC), Asthma, HIV, Malaria, Muscular Dystrophy and Sarcoidosis. Gene expression meta-analyses were undertaken in each disease independently, and significant differentially expressed genes (DEGs) were selected as those displaying a FDR corrected p-value (q-value)<0.05.
(XLS)

Table S2 Differentially Expressed Genes (DEGs, q-value<0.05) deregulated in opposite directions in CNS disorders and Cancers. The two first sheets correspond to the DEGs that are significantly deregulated in opposite direction concurrently in the three CNS disorders and the three Cancer types (as in **Table 1** and **Table 2**). The following two sheets list the DEGs that are deregulated in opposite direction in at least two CNS disorders and two Cancer types. Sheets number 5 and 6 show the DEGs deregulated in opposite directions in at least one CNS disorder and one Cancer type. The last four sheets enumerate the DEGs deregulated in opposite directions in at least two CNS disorders and one Cancer type and DEGs deregulated in opposite directions in at least one CNS disorder and two Cancer types.
(XLS)

Table S3 Pathways significantly up- and down-regulated in each CNS disorder and Cancer type after Gene set enrichment analysis (GSEA, q-value<0.05) considering KEGG, Reactome and Biocarta pathway databases.
(XLS)

Text S1 Workflow of the analysis pipeline and microarray expression datasets used in the meta-analyses.
(PDF)

Text S2 Dynamic report of the analysis pipeline.
(PDF)
