# Supplementary material for: Molecular Evidence for the Inverse Comorbidity between Central Nervous System Disorders and Cancers Detected by Transcriptomic Meta-analyses
Source: PLoS Genet. 2014 Feb 20;10(2):e1004173. doi: 10.1371/journal.pgen.1004173 (PMC3930576; doi:10.1371/journal.pgen.1004173)
Supplement: Figure S1 — Comparisons of Differentially Expressed Genes (DEGs) associated with Central Nervous System (CNS) disorders and Cancers at different q-value thresholds. The DEGs up- and down-regulated after gene expression meta-analysis in each CNS disorder (Alzheimer's Disease, AD; Parkinson's Disease, PD; and Schizophrenia, SCZ) and in each Cancer (Colorectal Cancer, CRC; Prostate Cancer, PC; Lung Cancer, LC) are selected for the 0.005 (a), 0.0005 (b), 0.00005 (c) and 0.000005 (d) thresholds, and compared to each others. (PDF) [file pgen.1004173.s001.pdf]

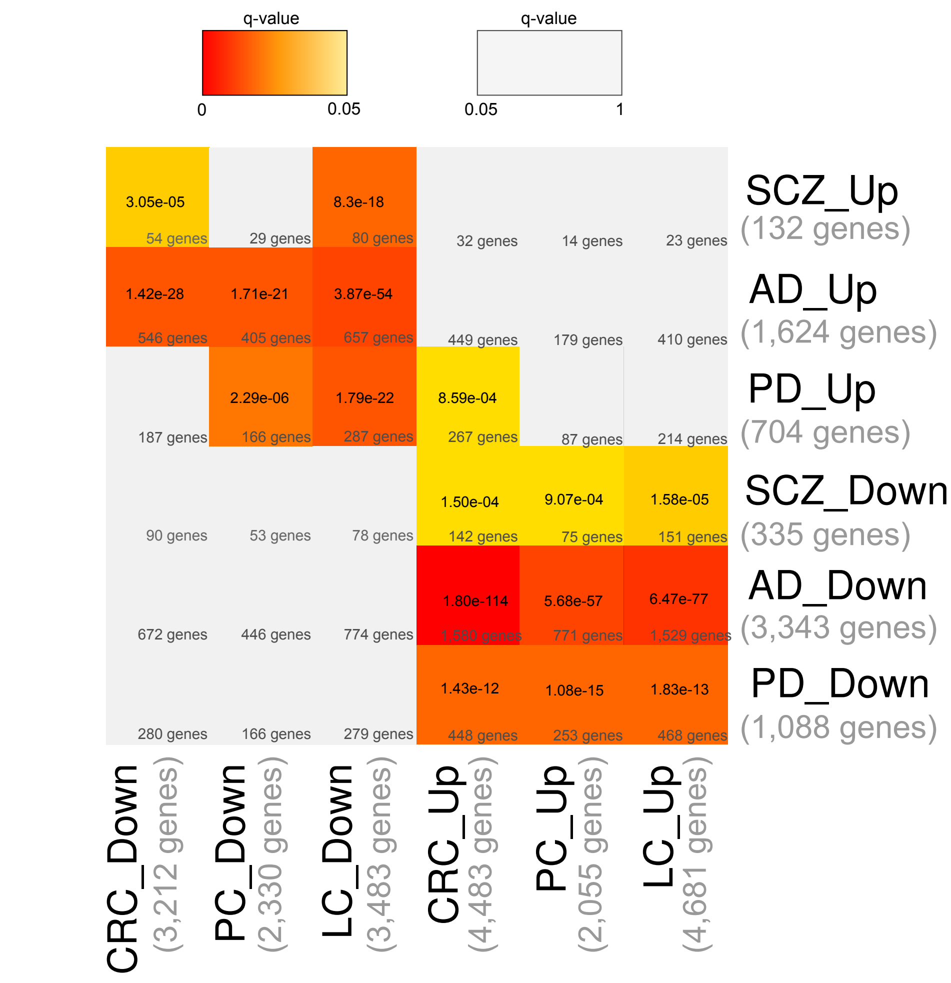

DEGs associated with CNS disorders and Cancers at **0.005**

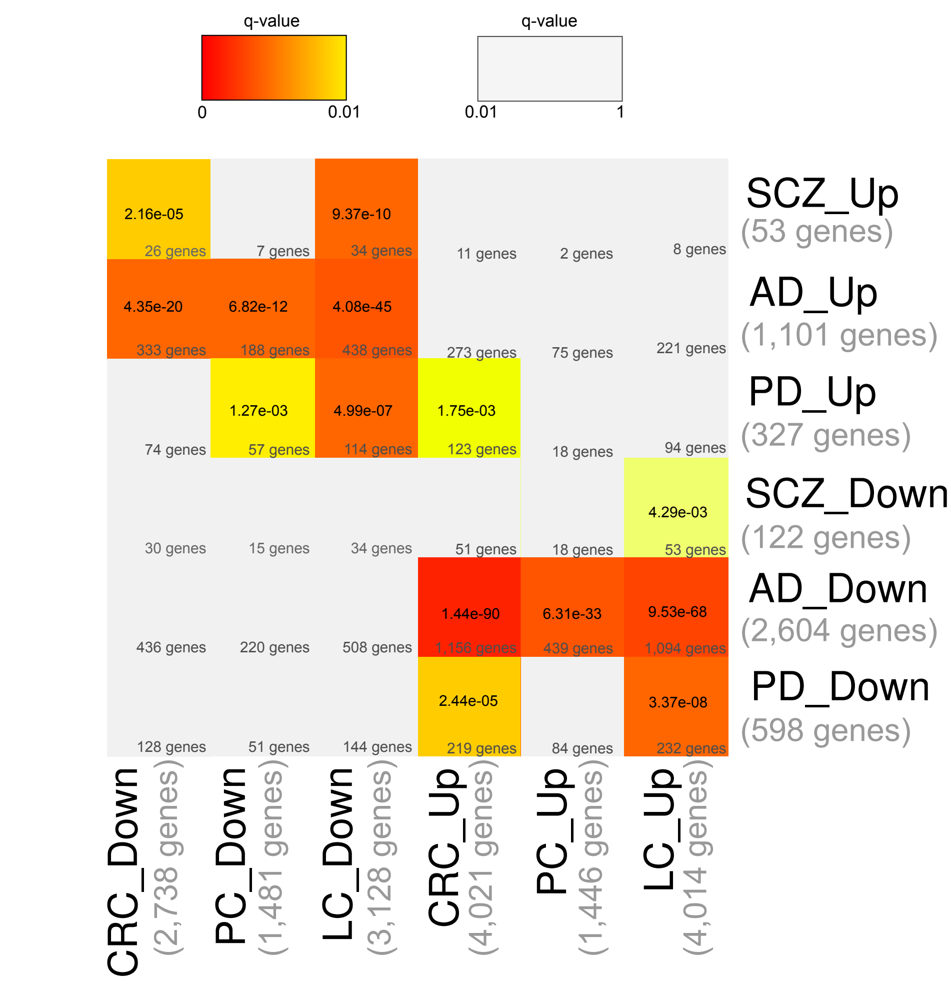

DEGs associated with CNS disorders and Cancers at **0.0005**

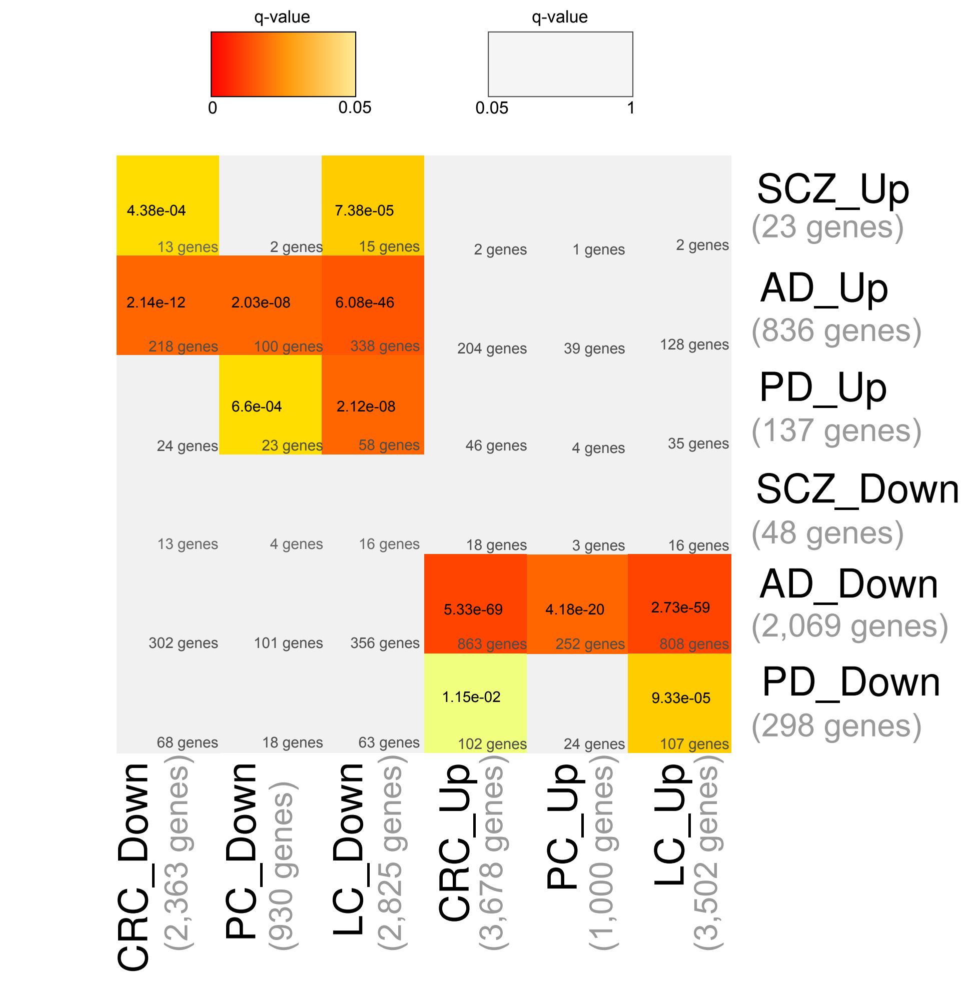

DEGs associated with CNS disorders and Cancers at **0.00005**

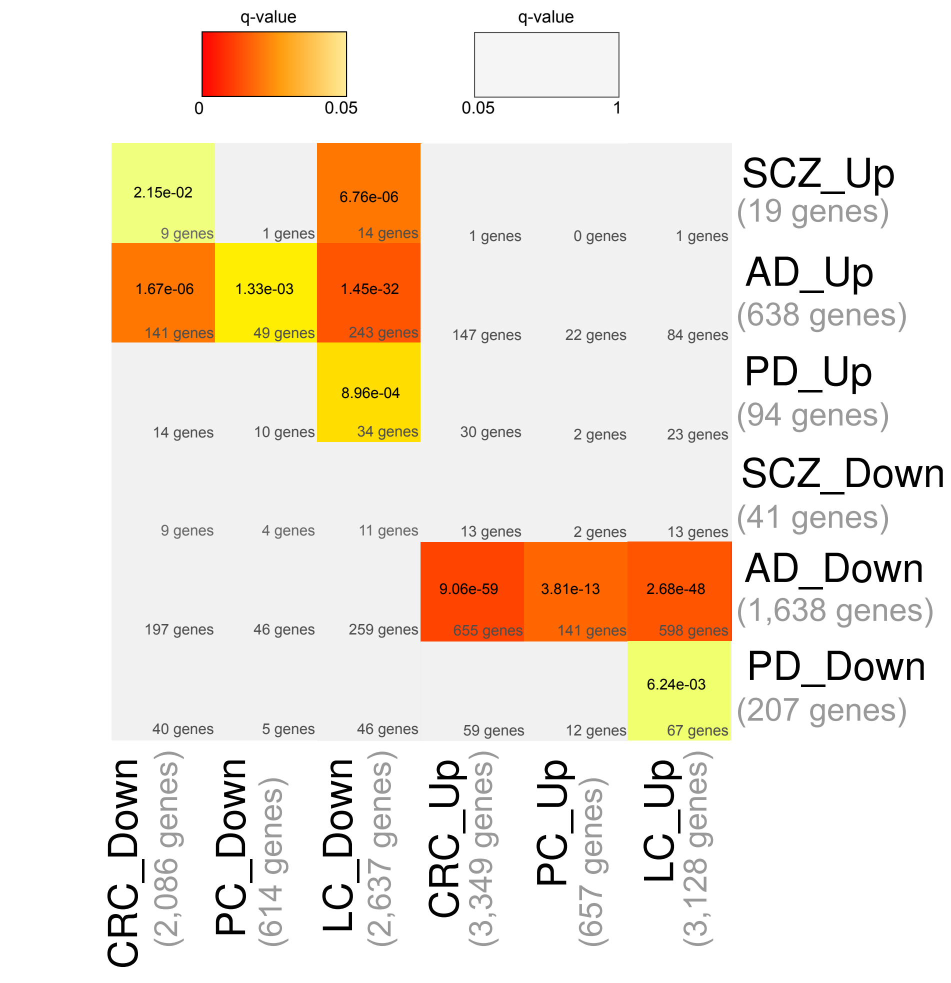

DEGs associated with CNS disorders and Cancers at **0.000005**
