# Supplementary figures and images for: Molecular Evidence for the Inverse Comorbidity between Central Nervous System Disorders and Cancers Detected by Transcriptomic Meta-analyses
Source: PLoS Genet. 2014 Feb 20;10(2):e1004173. doi: 10.1371/journal.pgen.1004173 (PMC3930576; doi:10.1371/journal.pgen.1004173)

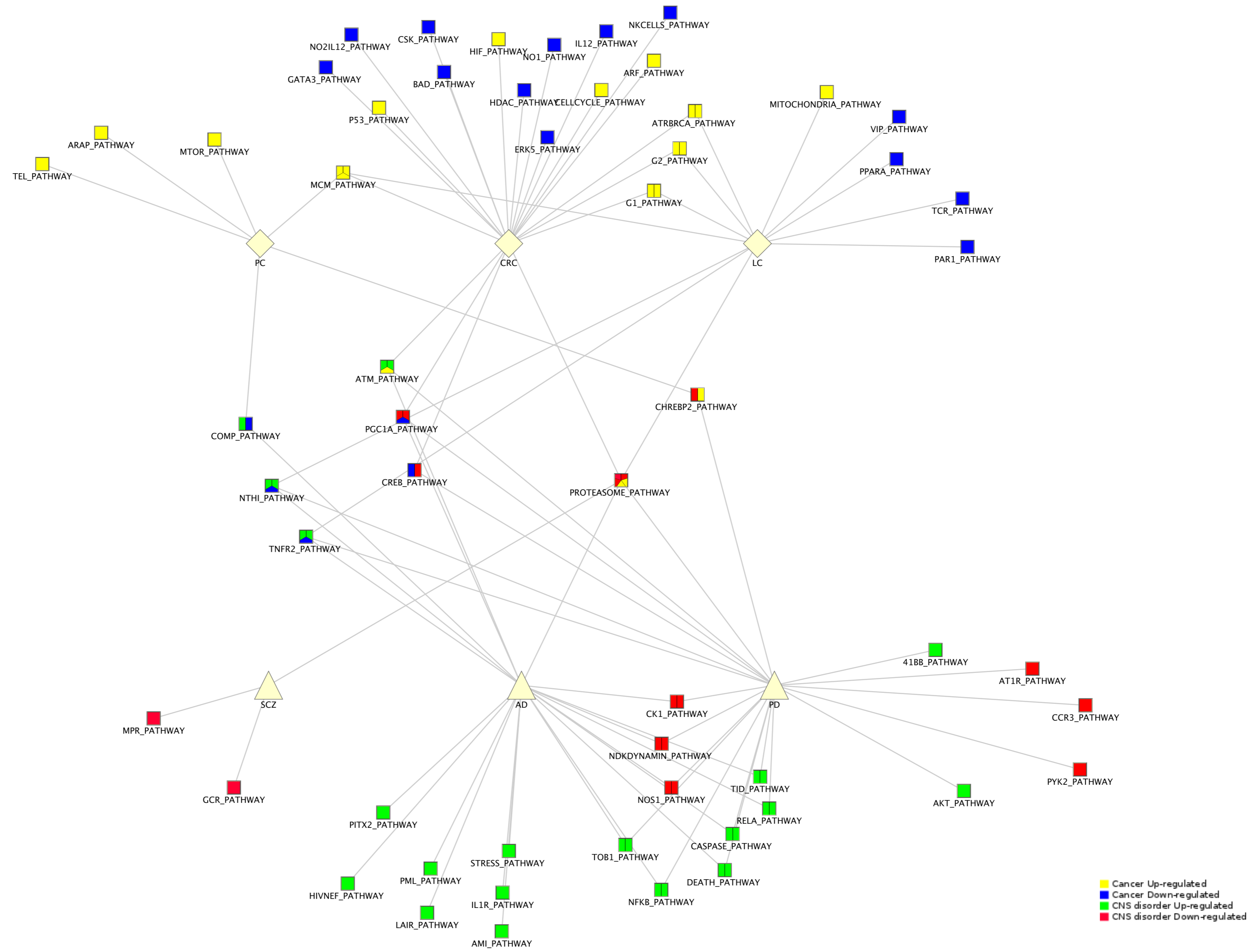

Biocarta

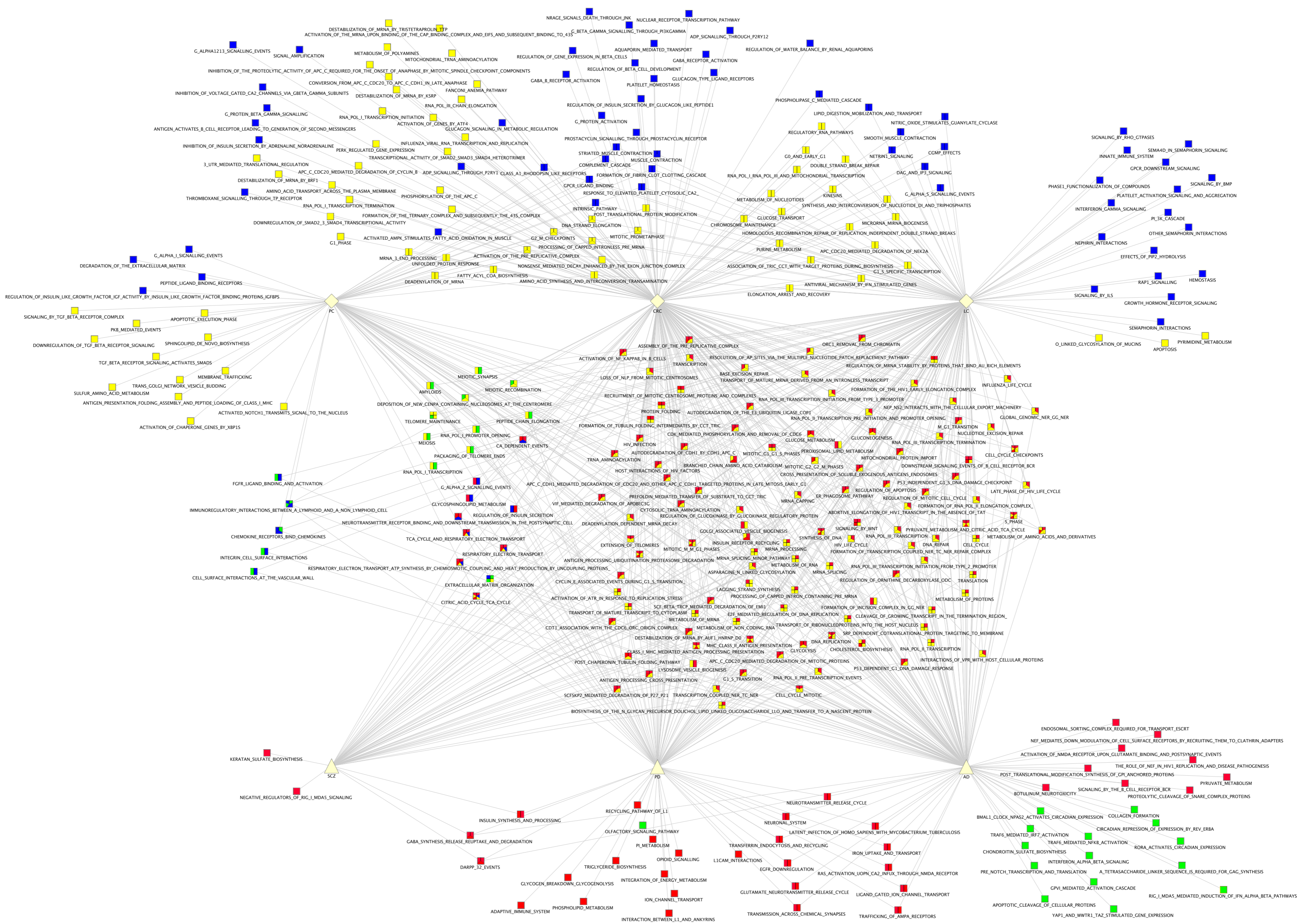

Reactome

Supplement: Figure S2 — Biocarta and Reactome pathways significantly deregulated in the three types of Cancers and CNS disorders. Biocarta pathways (http://www.biocarta.com/) and Reactome pathways [26] Cancer upregulated (yellow), Cancer downregulated (blue), CNS disorder upregulated (green) and CNS disorder downregulated (red). The green/blue and yellow/red associations thus correspond to pathways deregulated in opposite directions in CNS disorders and Cancers. All networks are available at bioinfo.cnio.es/people/cboullosa/validation/cytoscape/Ibanezetal.zip, in cytoscape format (http://www.cytoscape.org/). (PDF) [file pgen.1004173.s002.pdf]
