# Supplementary material for: Molecular Evidence for the Inverse Comorbidity between Central Nervous System Disorders and Cancers Detected by Transcriptomic Meta-analyses
Source: PLoS Genet. 2014 Feb 20;10(2):e1004173. doi: 10.1371/journal.pgen.1004173 (PMC3930576; doi:10.1371/journal.pgen.1004173)
Supplement: Text S1 — Workflow of the analysis pipeline and microarray expression datasets used in the meta-analyses. (PDF) [file pgen.1004173.s006.pdf]

Workflow

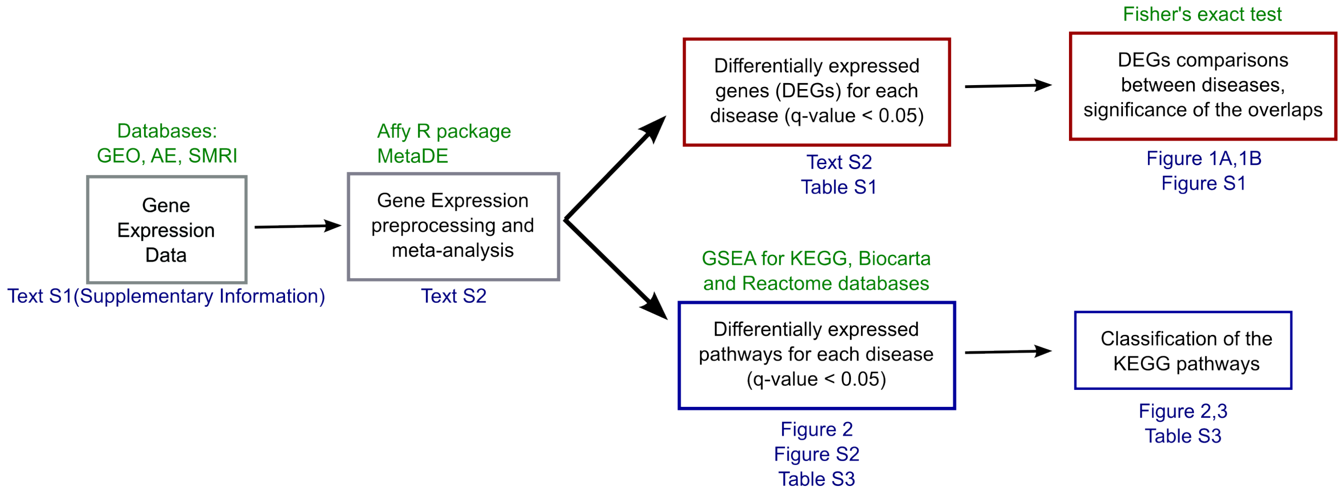
Databases:
GEO, AE, SMRI
Gene
Expression
Data
Text S1(Supplementary Information)
Affy R package
MetaDE
Gene Expression
preprocessing and
meta-analysis
Text S2
Differentially expressed
genes (DEGs) for each
disease (q-value < 0.05)
Text S2
Table S1
Fisher's exact test
DEGs comparisons
between diseases,
significance of the overlaps
Figure 1A,1B
Figure S1
GSEA for KEGG, Biocarta
and Reactome databases
Differentially expressed
pathways for each disease
(q-value < 0.05)
Figure 2
Figure S2
Table S3
Classification of the
KEGG pathways
Figure 2,3
Table S3

Supplementary Information

Gene expression datasets

Alzheimer's disease

● GSE5281 (http://www.ncbi.nlm.nih.gov/geo/query/acc.cgi?acc=GSE5281)

Entorhinal Cortex samples: 13 controls, 10 alzheimer

Hippocampus samples: 13 controls, 10 alzheimer

Medial Temporal Gyrus samples: 12 controls, 16 alzheimer

Posterior Singulate samples: 13 controls, 9 alzheimer

Primary Visual Cortex samples: 12 controls, 19 alzheimer

Superior Frontal Gyrus samples: 11 controls, 23 alzheimer

Platform: Affymetrix Human Genome U133 Plus 2.0 Array

● GSE1297 (http://www.ncbi.nlm.nih.gov/geo/query/acc.cgi?acc=GSE1297)

Hippocampus samples

9 controls

15 alzheimer (Severe and Moderate)

Platform: Affymetrix Human Genome U133 Plus 2.0 Array

Parkinson's disease

● GSE7621 (http://www.ncbi.nlm.nih.gov/geo/query/acc.cgi?acc=GSE7621)

Postmortem substantia nigra samples

9 controls

16 parkinson

Platform: Affymetrix Human Genome U133 Plus 2.0 Array

● GSE8397 (http://www.ncbi.nlm.nih.gov/geo/query/acc.cgi?acc=GSE8397)

Postmortem medial, lateral and frontal samples

16 controls

29 parkinson

Platform: Affymetrix Human Genome U133A Array

● GSE20292 (http://www.ncbi.nlm.nih.gov/geo/query/acc.cgi?acc=GSE20292)

Substantia nigra samples, age- and gender-matched controls

18 controls

11 parkinson

Platform: Affymetrix Human Genome U133A Array

Schizophrenia

● GSE4036 (http://www.ncbi.nlm.nih.gov/geo/query/acc.cgi?acc=GSE4036)28 postmortem cerebellar tissues from schizophrenia patients and sex-, age-, and PMI-matched controls.

14 schizophrenia

14 controls

Platform: Affymetrix Human Genome U133 Plus 2.0 Array

The following experiments were downloaded from the Stanley Medical Research Institute, Online Genomics Database (SMRI, https://www.stanleygenomics.org) that manage 2 collections of brain samples (the Stanley Array collection and the Stanley Consortium collection). The different studies have been conducted using overlapping subsets of these collections.

● Dobrin (https://www.stanleygenomics.org/stanley/standard/studyDetail.jsp?study_id=5)

postmortem frontalBA46 cortice samples

30 schizophrenia

25 controls

Platform: Affymetrix Human Genome U133 Plus 2.0 Array

● Laeng (https://www.stanleygenomics.org/stanley/standard/studyDetail.jsp?study_id=17)41 postmortem hippocampus (CA1)

20 schizophrenia

21 controls

Platform: Affymetrix Human Genome U133 Plus 2.0 Array

● Kemether (https://www.stanleygenomics.org/stanley/standard/studyDetail.jsp?study_id=16)26 postmortem thalamus (MD) samples

14 schizophrenia

12 controls

Platform: Affymetrix Human Genome U133 Plus 2.0 Array

● AltarA (https://www.stanleygenomics.org/stanley/standard/studyDetail.jsp?study_id=1)67 postmortem frontalBA46 cortice samples

33 schizophrenia

34 controls

Platform: Affymetrix Human Genome U133A Array

● AltarC (https://www.stanleygenomics.org/stanley/standard/studyDetail.jsp?study_id=2)50 postmortem frontalBA46 cortice samples

21 schizophrenia

29 controls

Platform: Affymetrix Human Genome U133A Array

● Bahn (https://www.stanleygenomics.org/stanley/standard/studyDetail.jsp?study_id=3)67 postmortem frontalBA46 cortice samples

34 schizophrenia

33 controls

Platform: Affymetrix Human Genome U133A Array

● Kato (https://www.stanleygenomics.org/stanley/standard/studyDetail.jsp?study_id=7)69 postmortem frontalBA46 cortice samples

35 schizophrenia

34 controls

Platform: Affymetrix Human Genome U133A Array

Colorectal cancer

● Skrzypczak (GSE20916) (http://www.ncbi.nlm.nih.gov/geo/query/acc.cgi?acc=GSE20916)

Macro- and micro- dissected samples

81 cases

24 controls

Platform: Affymetrix Human Genome U133 Plus 2.0 Array

● Sabates-Bellver (GSE8671) (http://www.ncbi.nlm.nih.gov/geo/query/acc.cgi?acc=GSE8671)

32 cases

32 controls

Platform: Affymetrix Human Genome U133 Plus 2.0 Array

● Hong (GSE9348) (http://www.ncbi.nlm.nih.gov/geo/query/acc.cgi?acc=GSE9348)

69 cases

12 controls

Platform: Affymetrix Human Genome U133 Plus 2.0 Array

- GSE4183 (http://www.ncbi.nlm.nih.gov/geo/query/acc.cgi?acc=GSE4183), GSE7307 (GSM175905) (http://www.ncbi.nlm.nih.gov/geo/query/acc.cgi?acc=GSM175905), GSE2109 (http://www.ncbi.nlm.nih.gov/geo/query/acc.cgi?acc=GSE2109)

24 cases

9 control

Platform: Affymetrix Human Genome U133 Plus 2.0 Array

Lung cancer

● GSE3526 + GSE19188 (http://www.ncbi.nlm.nih.gov/geo/query/acc.cgi?acc=GSE3526 + http://www.ncbi.nlm.nih.gov/geo/query/acc.cgi?acc=GSE19188)

68 controls

110 cases

Platform: Affymetrix Human Genome U133 Plus 2.0 Array

● GSE7670 (*http://www.ncbi.nlm.nih.gov/geo/query* /acc.cgi?acc=GSE7670)

27 controls

27 cases

Platform: Affymetrix Human Genome U133A Array

● GSE10072 (http://www.ncbi.nlm.nih.gov/geo/query/acc.cgi?acc=GSE10072)

49 control

58 cases

Platform: Affymetrix Human Genome U133A Array

Prostate cancer

● GSE17951 (http://www.ncbi.nlm.nih.gov/geo/query/acc.cgi?acc=GSE17951)

Gene expression analysis of prostate cancer samples using Affymetrix U133Plus2 array

13 control

109 cases

Platform: Affymetrix Human Genome U133 Plus 2.0 Array

● E-TABM-26 (http://www.ebi.ac.uk/arrayexpress/experiments/E-TABM-26/)

Transcription profiling of human prostate tissues obtained from multiple Institutions

13 control

44 cases

Platform: Affymetrix Human Genome U133A Array

● GSE6956 (http://www.ncbi.nlm.nih.gov/geo/query/acc.cgi?acc=GSE6956)

Transcription profiling of human prostate tissues

18 control

71 cases

Platform: Affymetrix Human Genome U133A 2.0 Array

HIV

● GSE16363 (http://www.ncbi.nlm.nih.gov/geo/query/acc.cgi?acc=GSE16363)

Microarray Analysis of Lymphatic Tissue Reveals Stage-Specific, Gene-Expression Signatures in HIV- 1 Infection Tissue: lymph node

10 control

24 HIV

Platform: Affymetrix Human Genome U133 Plus 2.0 Array

● GSE28160 (http://www.ncbi.nlm.nih.gov/geo/query/acc.cgi?acc=GSE28160)

Gene Expression in Brain Tissues of Patients with HIV-Associated Neurocognitive Disorders Tissue: centrum semiovale (deep white matter), postmortem

9 control

14 HIV

Platform: Affymetrix Human Genome U133 Plus 2.0 Array

Malaria

● GSE5418 (http://www.ncbi.nlm.nih.gov/geo/query/acc.cgi?acc=GSE5418)

Gene Expression Analysis in Malaria Infection. Tissue: peripheral blood mononuclear cells

14 control samples

22 malaria samples

Platform: Affymetrix Human Genome U133A Array

Muscular Dystrophy

● GSE6011 (http://www.ncbi.nlm.nih.gov/geo/query/acc.cgi?acc=GSE6011)

Expression data from quadriceps muscle of young Duchenne muscular dystrophy (DMD) patients and age matched controls. Tissue: quadriceps muscle

14 control samples

23 muscular dystrophy

Platform: Affymetrix Human Genome U133A Array

● GSE11681 (http://www.ncbi.nlm.nih.gov/geo/query/acc.cgi?acc=GSE11681)

Tissue: quadriceps and biceps muscles

10 controls

10 limb-girdle muscular dystrophy (LGMD) type 2A samples

Platform: Affymetrix Human Genome U133A Array

Pulmonar Sarcoidosis

● GSE16538 (http://www.ncbi.nlm.nih.gov/geo/query/acc.cgi?acc=GSE16538)

Genome-wide gene expression profile analysis in pulmonary sarcoidosis. Tissue: lung

6 control samples

6 sarcoidosis samples

Platform: Affymetrix Human Genome U133 Plus 2.0 Array

Asthma

● GSE31773 (http://www.ncbi.nlm.nih.gov/geo/query/acc.cgi?acc=GSE31773)

Comparison of mRNA expression in circulating T-cells from patients with severe asthma Tissue: circulating CD4+ and CD8+ T-cells

16 controls

24 asthma

Platform: Affymetrix Human Genome U133 Plus 2.0 Array

- GSE22528 (http://www.ncbi.nlm.nih.gov/geo/query/acc.cgi?acc=GSE22528)

Gene expression pattern of alveolar macrophages of allergic asthmatics in comparison with control subjects Tissue: alveolar macrophage samples from bronchoalveolar lavages

5 controls

5 asthma

Platform: Affymetrix Human Genome U133A Array
