# Supplementary material for: Molecular Evidence for the Inverse Comorbidity between Central Nervous System Disorders and Cancers Detected by Transcriptomic Meta-analyses
Source: PLoS Genet. 2014 Feb 20;10(2):e1004173. doi: 10.1371/journal.pgen.1004173 (PMC3930576; doi:10.1371/journal.pgen.1004173)
Supplement: Text S2 — Dynamic report of the analysis pipeline. (PDF) [file pgen.1004173.s007.pdf]

Molecular Evidence for the Inverse Comorbidity between Central Nervous System Disorders and Cancers detected by Transcriptomic Meta-analyses

Ibáñez K, Boullosa C, Tabarés-Seisdedos R, Baudot A and Valencia A

Details and Code for the Analysis

The KnitR package (http://yihui.name/knitr/) is a simple text-markup language called markdown to write reproducible reports of analysis workflow, providing full transparency and reproducibility.

Our analysis workflow (Text S1) is divided into 3 parts: Part1) Microarray data normalization, Part2) Meta-analysis for the identification of significantly Differentially Expressed Genes (DEGs) in each disease and Part3) Fisher's exact test for the comparison of DEGs between the different diseases. This is the initial look:

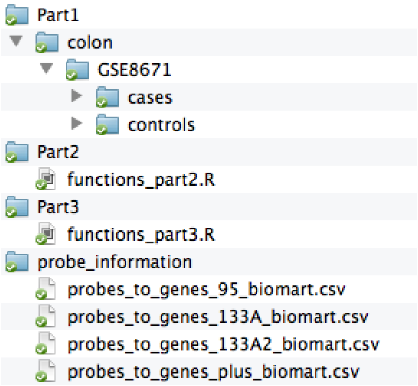

The files named as "probes_to_genes_NAMEofPLATFORM_biomart.csv"(NAMEofPLATFORM being *plus* for Chip® Human Genome U133Plus 2.0, *133A* for Chip® Human Genome U133A and *133A2* for Chip® Human Genome U133A2) contain probes to genes mapping information, extracted from BioMart (http://www.ensembl.org/info/data/biomart.html).

Part 1. Normalization of the raw microarray expression data (CEL files)

Gene expression raw data (CEL files) have been downloaded from NCBI GEO omnibus, EBI ArrayExpress and SMRI (Text S2), we do applied the normalization procedure using for that frozen Robust Multiarray Analysis (fRMA) from the R Affy package.

We are showing here as example the normalization of the gene expression dataset GSE8671 (Sabates-Bellver J. *et al.*), that profiles gene expression in CRC. Notice that two folders are created for each study: *controls* and *cases*.

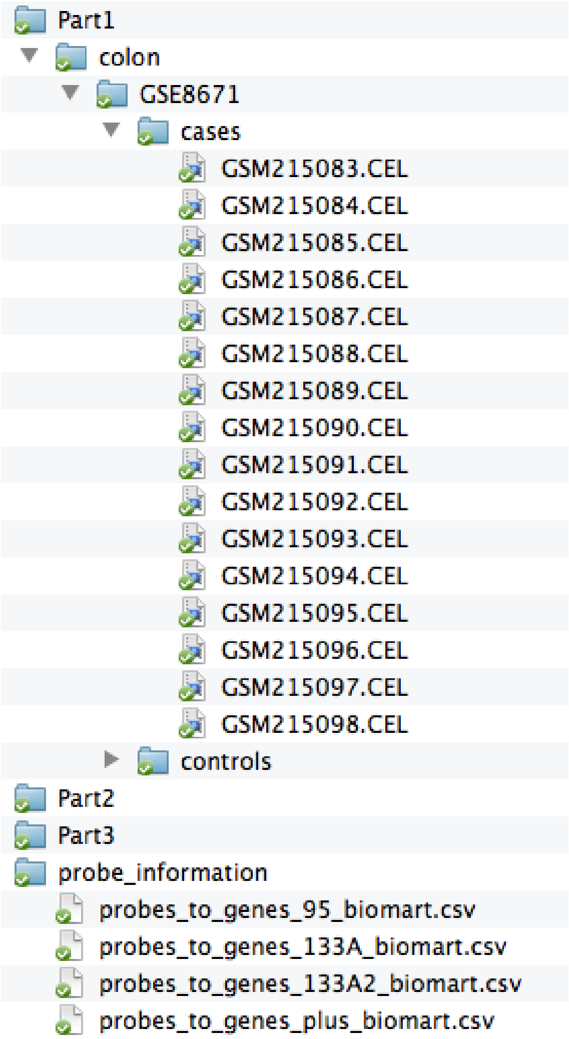

The Part1 data files presented here contain only one study. In the original application of the workflow, they contain all studies for all diseases.

The R code for the normalization part is the following:

```
# may install the following packages:

# source('http://www.bioconductor.org/biocLite.R') biocLite('affy')
# biocLite('frma') biocLite('hgu133plus2frmavecs')

# Load the corresponding libraries
library(affy)
library(frma)

# Download gene expression datasets from GEO In this example:
# http://www.ncbi.nlm.nih.gov/geo/query/acc.cgi?acc=GSE8671 In
# Part1/colon/GSE8671 colon CEL files are divided into 'cases' and
# 'controls' We do a separation between cases and controls after downloading
# CEL files (take a look in each gene expression study's metadata)
```

```r
affyDir <- "Part1/colon/GSE8671"
list <- c("cases/", "controls/")
FileName <- c()
Class <- c()

# Creation of a data.frame with each CEL file names (FileName field) and 1
# if the CEL file is a case sample or 0 if it is a control sample (Class
# field)
targets <- data.frame()
for (k in 1:length(list)) {
    affyDir_path <- paste(affyDir, list[k], sep = "/")
    listFiles <- list.files(affyDir_path)
    FileName <- c(FileName, paste(affyDir_path, listFiles, sep = ""))
    count <- rep(2 - k, length(listFiles))
    Class <- c(Class, count)
}
targets <- data.frame(FileName, Class)


head(targets)
```

```
##                                    FileName Class
## 1 Part1/colon/GSE8671/cases/GSM215083.CEL     1
## 2 Part1/colon/GSE8671/cases/GSM215084.CEL     1
## 3 Part1/colon/GSE8671/cases/GSM215085.CEL     1
## 4 Part1/colon/GSE8671/cases/GSM215086.CEL     1
## 5 Part1/colon/GSE8671/cases/GSM215087.CEL     1
## 6 Part1/colon/GSE8671/cases/GSM215088.CEL     1
```

```r
tail(targets)
```

```
##                                        FileName Class
## 59 Part1/colon/GSE8671/controls/GSM215077.CEL     0
## 60 Part1/colon/GSE8671/controls/GSM215078.CEL     0
## 61 Part1/colon/GSE8671/controls/GSM215079.CEL     0
## 62 Part1/colon/GSE8671/controls/GSM215080.CEL     0
## 63 Part1/colon/GSE8671/controls/GSM215081.CEL     0
## 64 Part1/colon/GSE8671/controls/GSM215082.CEL     0
```

```r
# Creation of affyBatch object: ReadAffy reads CEL files into an Affybatch
# object Required to do the background correction and normalization with
# fRMA
affyBatch <- ReadAffy(filenames = as.character(targets$FileName))

# Background correction with fRMA and quantiles normalizations
expSet <- frma(affyBatch)

# Convert expSet to a data.frame and add headers
df <- as.data.frame(exprs(expSet))

header.df <- gsub(1, "Case", targets$Class)
header.df <- gsub(0, "Control", header.df)
colnames(df) <- header.df
```

```r
# Mapping of probes to gene symbols
biomart <- read.csv2(file = "probe_information/probes_to_genes_plus_biomart.csv",
    sep = "\t", header = F)

# Add a column called 'SYMBOL' that represents the gene symbols that capture
# the different probes The row called 'label' represents in numeric whether
# the sample is a case (1) or a control (1) Necessary in the following
# parts.
index_probes <- pmatch(as.character(rownames(df)), biomart$V1)
list_probes <- as.character(biomart$V2[index_probes])
list_probes <- c("NA", list_probes)
df <- rbind(as.integer(targets$Class), df)
df <- cbind(list_probes, df)
rownames(df)[1] <- "label"
colnames(df)[1] <- "SYMBOL"
head(df)
```

```
##            SYMBOL  Case  Case  Case  Case  Case  Case   Case  Case  Case
## label          NA 1.000 1.000 1.000 1.000 1.000 1.000  1.000 1.000 1.000
## 1007_s_at    DDR1 9.974 9.855 9.512 9.514 9.480 9.680 10.000 9.664 9.541
## 1053_at      RFC2 7.377 6.961 7.449 7.860 7.676 6.757  7.056 7.686 7.158
## 117_at      HSPA6 5.692 5.231 5.530 5.575 5.190 5.820  5.400 5.625 5.339
## 121_at       PAX8 8.124 7.629 7.610 7.833 7.348 8.104  8.019 8.220 8.225
## 1255_g_at GUCA1A 3.582 3.491 3.545 3.633 3.380 3.586  3.375 3.534 3.556
##             Case   Case  Case  Case  Case  Case  Case  Case  Case  Case
## label      1.000  1.000 1.000 1.000 1.000 1.000 1.000 1.000 1.000 1.000
## 1007_s_at 9.718 10.144 9.450 9.674 9.689 9.483 9.306 9.553 9.602 9.887
## 1053_at   7.678  6.174 7.768 7.212 8.059 7.464 7.570 8.097 7.369 7.190
## 117_at    5.459  6.340 5.413 5.434 5.421 5.567 5.242 5.603 5.183 5.578
## 121_at    7.886  8.139 7.453 7.541 7.973 8.108 7.918 7.823 8.343 8.173
## 1255_g_at 3.566  3.716 3.503 3.415 3.542 3.509 3.482 3.451 3.549 3.779
##              Case  Case  Case  Case  Case  Case  Case   Case  Case  Case
## label       1.000 1.000 1.000 1.000 1.000 1.000 1.000  1.000 1.000 1.000
## 1007_s_at 10.185 9.648 9.707 9.630 9.515 9.266 9.800 10.067 9.715 9.778
## 1053_at    7.052 7.081 7.793 6.905 7.817 8.040 7.022  6.849 7.456 7.255
## 117_at     5.507 5.291 5.312 5.463 5.393 5.289 5.401  5.212 5.302 5.621
## 121_at     8.262 7.767 8.121 7.567 7.764 7.704 7.953  7.892 8.054 7.654
## 1255_g_at  3.469 3.614 3.494 3.338 3.366 3.631 3.670  3.604 3.413 3.457
##             Case  Case  Case Control Control Control Control Control
## label      1.000 1.000 1.000   0.000   0.000   0.000   0.000   0.000
## 1007_s_at 9.669 9.856 9.861  10.000   9.915   9.765   9.696   9.621
## 1053_at   7.655 8.413 7.170   6.294   6.425   6.242   5.888   6.504
## 117_at    5.679 5.238 5.493   6.006   5.447   5.708   5.537   5.493
## 121_at    7.590 7.490 7.947   8.351   7.925   7.854   7.947   7.881
## 1255_g_at 3.373 3.497 3.437   3.393   3.600   3.565   3.740   3.457
##           Control Control Control Control Control Control Control Control
## label       0.000   0.000   0.000   0.000   0.000   0.000   0.000   0.000
## 1007_s_at   9.832   9.721   9.685   9.601   9.822   9.306   9.734   9.667
## 1053_at     6.166   6.449   6.611   6.457   6.172   6.427   6.741   6.431
## 117_at      5.690   5.818   5.757   5.720   5.788   5.256   5.573   6.643
## 121_at      8.060   7.919   8.029   8.087   8.006   7.763   7.566   7.740
## 1255_g_at   3.448   3.468   3.417   3.422   3.662   3.479   3.419   3.559
##           Control Control Control Control Control Control Control Control
## label       0.000   0.000   0.000   0.000   0.000   0.000   0.000   0.000
```

```
## 1007_s_at   9.619   9.617   9.704   9.683   9.941   9.853   9.867   9.780
## 1053_at     6.791   6.496   6.350   6.658   6.421   6.542   6.123   6.147
## 117_at      5.650   5.639   5.372   5.995   5.558   5.530   5.505   5.443
## 121_at      8.081   7.928   7.930   8.134   8.264   7.803   8.035   7.805
## 1255_g_at   3.301   3.585   3.384   3.547   3.474   3.421   3.456   3.585
##           Control Control Control Control Control Control Control Control
## label       0.000   0.000   0.000   0.000   0.000   0.000   0.000   0.000
## 1007_s_at   9.541   9.764   9.790   9.919   9.617   9.706   9.804   9.910
## 1053_at     7.119   6.134   6.282   6.553   6.260   6.180   5.935   6.351
## 117_at      5.503   5.614   5.677   5.608   5.536   5.425   5.566   5.516
## 121_at      8.020   7.857   8.002   7.920   8.271   8.052   8.095   7.799
## 1255_g_at   3.463   3.608   3.391   3.673   3.534   3.632   3.461   3.358
##           Control Control Control
## label       0.000   0.000   0.000
## 1007_s_at   9.898   9.830  10.057
## 1053_at     6.547   6.555   6.256
## 117_at      5.611   5.661   5.580
## 121_at      7.794   7.732   8.158
## 1255_g_at   3.531   3.547   3.428

# Write the output file, that will be taken as an input for the Part2
# (Meta-analysis)
output_fileName = "normalizationColon_sabatesvsControles_meta.txt"
output_dirName = "Part2/data/colon"
file_dir = paste(output_dirName, output_fileName, sep = "/")
write.table(df, file = file_dir, quote = F, row.names = T, col.names = T, sep = "\t")
```

The complete procedure of normalization exemplified here was then repeated for all studies in all diseases (CRC, LC and PC in cancers, AD, PD and SCZ in CNS disorders and Asthma, HIV, Malaria, Dystrophy and Sarcoidosis). For each study in each disease, the normalization phase creates in Part2/data the files ready to be analyzed with MetaDE.

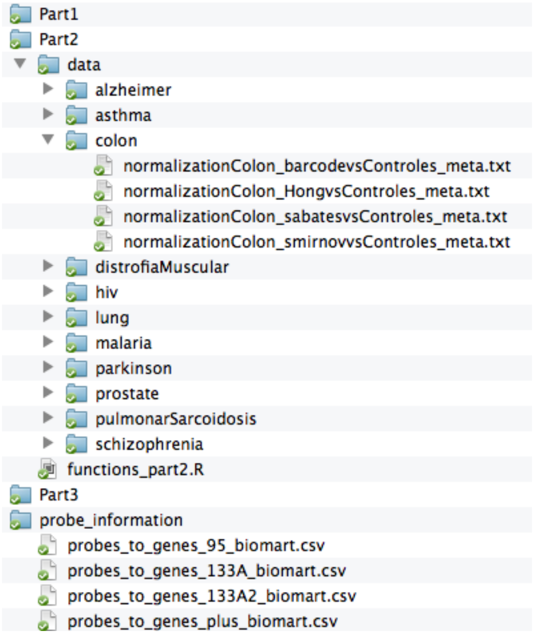

Part 2. Meta-analysis (MetaDE)

For each disease, the normalized data from the different studies are now stored in Part2/data. We now apply the meta-analysis thanks to the MetaDE R package, using the Fixed Effect Model method. This method is widely used to compute meta-analyses for microarray data (Choi *et al.* 2003, Tseng *et al.* 2012). The main methods we call are contained in Part2/functions_part2.R.

Before doing the meta-analysis for each disease, the data must be preprocessed to assess the effect sizes and variances. Notice that the other meta-analysis method, such as Fisher, maxP or roP, would have necessitate to compute the DEGs for each individual study, instead of assessing the effect sizes and variances of all studies, and then compute the DEGs only once.

The method *various.effect.sizes* which uses MetaDE's *ind.cal.ES* function, estimates the effect sizes and variances for each disease (output in *intermediate_data/Effects&Variances*) so as to run a metaanalysis with Fixed Effects Model (FEM) (output in "MetaDE_output"). This function uses as input the MetaDE.filter function's output (contained in *./Part2/intermediate_data/individual_filtered (MetaDE.filter)*). The implemented code is the following:

```
# may install the following packages:

# source('http://bioconductor.org/biocLite.R') biocLite('impute')
# biocLite('Biobase') install.packages('MetaDE') install.packages('MetaQC')
# biocLite('limma') biocLite('AnnotationDbi') biocLite('genefilter')
# biocLite('GSEABase') biocLite('qvalue') install.packages('MetaPath')
# install.packages('MetaDE')


library(MetaDE)

# Creation of a folder that will contain intermediate data
dir.aux <- "./Part2/intermediate_data/"

# Must load the code file 'funciones.R' in order to call internal functions
source("./Part2/functions_part2.R")

nombres.dir <- c("alzheimer", "asthma", "colon", "distrofiaMuscular", "lung",
    "parkinson", "prostate", "schizophrenia", "hiv", "pulmonarSarcoidosis",
    "malaria")
directorios <- paste("./Part2/data", nombres.dir, sep = "/")
names(directorios) <- nombres.dir

generar_directorios(dir.aux)

posiciones.por.hacer <- 1:length(names(directorios))

# In preprocesar.lista : for each disease, data is matched (MetaDE.match),
# merged (MetaDE.merge) and filtered (MetaDE.filter) from the normalized
# data

# INPUT: normalized data (part 1), located in ./Part2/data/disease.name (in
# file 'data.zip'), where disease.name could be 'alzheimer', 'asthma',
# 'breast', 'colon', 'distrofiaMuscular', 'lung', 'parkinson', 'prostate',
# 'schizophrenia', 'hiv', 'pulmonarSarcoidosis' and 'malaria'. Each folder
```

```
# contains the normalization outputs from every different study into
# diseases.

# OUTPUT: It generates .Rdata files for each disease :'./Part2/intermediate_
# data/individual_reads' (MetaDE.read), './Part2/intermediate_
# data/individual_filtered' (MetaDE.filter), './Part2/intermediate_
# data/individual_matched' (MetaDE.match) and './Part2/intermediate_
# data/individual_merged' (MetaDE.merge)

for (i in 1:length(names(directorios))) {
    preprocesar.lista(directorios[i], dir.aux, p.rep = "IQR", formato = "txt",
        skip = 2, log2 = T, del.perc = c(0, 0), mvperc = 0)
}

## Load the previously generated data (from MetaDE matching, merging and
## filtering)
o <- cargar.preprocesado(names(directorios), dir.aux)

matcheados <- o$matcheados
mergeados <- o$mergeados
filtrados <- o$filtrados


# PREPROCESSING FOR METAANALYSIS: EFFECT SIZES & VARIANCES

# INPUT: data from MetaDE preprocessing data (filtered data) OUTPUT: in
# './Part2/intermediate_data/Effects&Variances', an Rdata for each disease
# contains the effect sizes and variances for all the studies of that
# disease

for (pos in posiciones.por.hacer) {
    nam <- names(directorios)[pos]
    # it calls to MetaDE 'ind.cal.ES' function
    kk <- various.effect.sizes(filtrados, nam)
}
```

At this level, the look of the files would be the following:

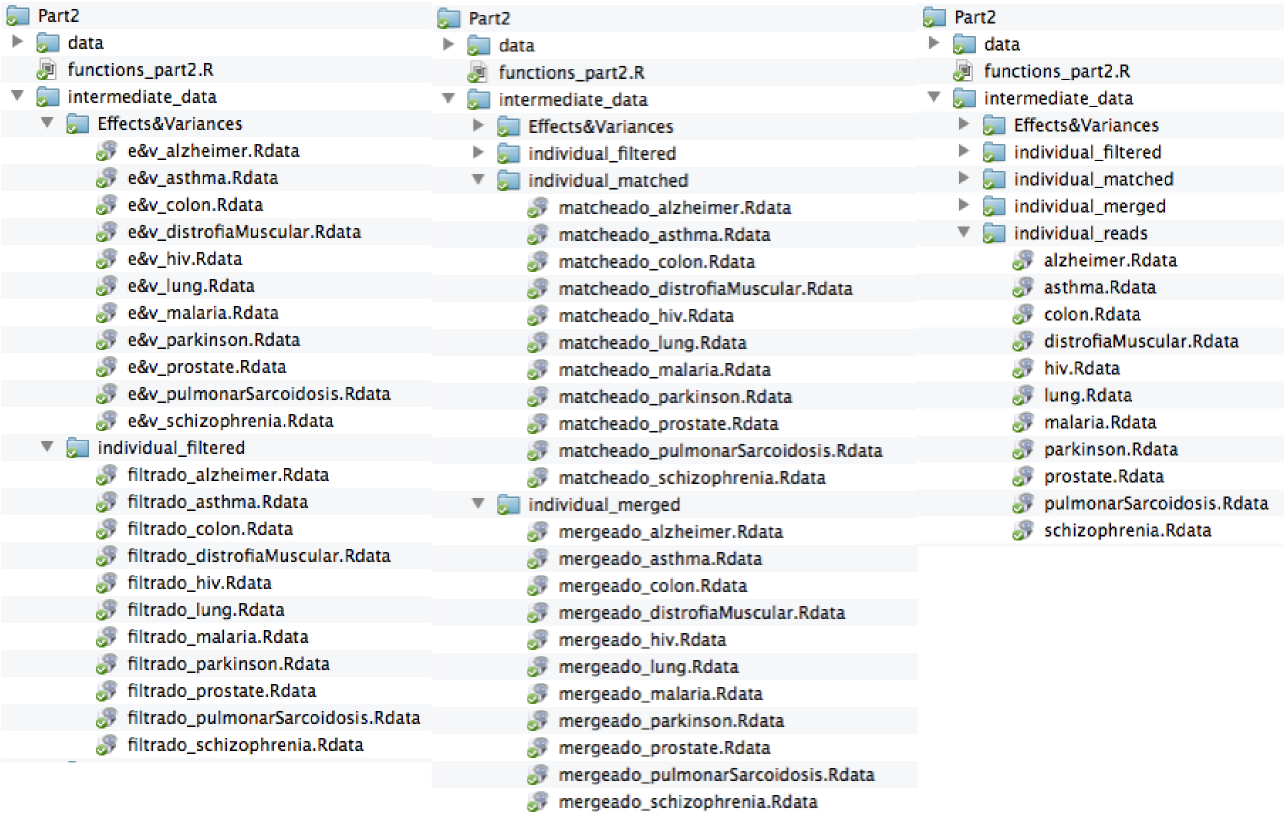

When we assess the effect sizes and variances of the data (MetaDE's *ind.cal.ES* function), necessary for the meta-analysis part, we can proceed with the FEM methodology.

```
# Load data from effect sizes and variances previously obtained
ves <- cargar.ves(names(directorios), dir.aux)

# Metaanalysis with the Fixed Effect Model (FEM)

# For each individual disease
results.FEM <- list()
for (pos in posiciones.por.hacer) {
    nam <- names(directorios)[pos]
    # Fixed effects model
    results.FEM[[nam]] <- FEM(ves, nam)
}

# Genes with corrected p-value (FDR) lower than a threshold (in our main
# analysis 0.05) are written into two different files, for up-regulated
# genes and down-regulated ones

# OUTPUT: In ./Part2/MetaDE_output/diseaseName_up_FEM and
# ./Part2/MetaDE_output/diseaseName_down_FEM are generated (the same output
# is also generated in a Rdata file in
# ./Part2/intermediate_data/meta/diseaseName_FEM.Rdata)

for (pos in posiciones.por.hacer) {
    nam <- names(directorios)[pos]
    load(paste(dir.aux, "meta/", nam, "_FEM.Rdata", sep = ""))
```

```
    up <- names(fem.out$FDR[(fem.out$FDR < 0.05) & (fem.out$zval > 0), ])
    down <- names(fem.out$FDR[(fem.out$FDR < 0.05) & (fem.out$zval < 0), ])
    write.table(up, file = paste("./Part2/MetaDE_output/", nam, "_up_FEM", sep = ""),
        quote = F, col.names = F, row.names = F)
    write.table(down, file = paste("./Part2/MetaDE_output/", nam, "_down_FEM",
        sep = ""), quote = F, col.names = F, row.names = F)
}
```

At the end of the meta-analysis, we will have the output up- and down-regulated DEGs for each diseases (Part2/MetaDE_output).

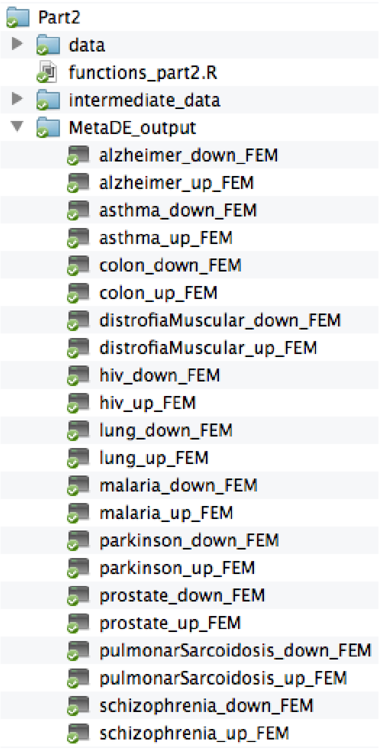

Part 3. Comparison of the DEGs of the different diseases. Fisher's Exact Test

The DEGs of each CNS disorder are compared to the DEGs of each Cancer type, and the significance of the overlaps between the DEGs are assessed by a one-tailed Fisher's exact test, corrected for multiple testing by the Bonferroni method. As we have studies from two different platforms (and one contains the other), in order to make the comparison between DEGs from different diseases (that can be obtained using different platforms), we have restricted the DEGs to the ones in common between both platforms, *i.e.*, the ones included in the smallest platform (GeneChip® Human Genome U133A). Thus, we used 14,538 (size of the U133A platform) as the background number of genes necessary for the Fisher's test

```
# Probe directory
dir.sondas2genes <- paste("./probe_information/", sep = "/")

# Directory if the outputs of the meta-analysis
dir.listas <- "./Part2/MetaDE_output"

# Directory of the final results
dir.out <- "./Part3/outputs"

columna.pvalue <- "adj.P.Val"

p.val <- 0.05
q.values <- p.val

source("./Part3/functions_part3.R")

# Creates the 'outputs' folder if it does not exist
crear.directorios(dir.out)

# Homogeneization of the gene symbols obtained from the different platforms
z <- obtener.probes2genes(dir.sondas2genes)
probes2genes.u95 <- z$u95
probes2genes.133A <- z[["133A"]]
probes2genes.133A2 <- z[["133A2"]]
probes2genes.plus <- z$plus

L <- obtener.listas(direct = dir.listas, type = "FEM")
names(L)

##  [1] "core.alzh.up"          "core.alzh.down"
##  [3] "core.park.up"          "core.park.down"
##  [5] "core.szo.up"           "core.szo.down"
##  [7] "core.lung.up"          "core.lung.down"
##  [9] "core.prostate.up"      "core.prostate.down"
## [11] "core.crc.up"           "core.crc.down"
## [13] "core.asthma.up"        "core.asthma.down"
## [15] "core.hiv.up"           "core.hiv.down"
## [17] "core.malaria.up"       "core.malaria.down"
## [19] "core.distrofia.up"     "core.distrofia.down"
## [21] "core.sarcoidosis.up"   "core.sarcoidosis.down"

if (!exists("L")) {
    L <- list()
}
```

```r
attach(L)

canceres.up <- c("core.lung.up", "core.prostate.up", "core.crc.up")
canceres.down <- c("core.lung.down", "core.prostate.down", "core.crc.down")
neuros.up <- c("core.szo.up", "core.alzh.up", "core.park.up")
neuros.down <- c("core.szo.down", "core.alzh.down", "core.park.down")


# FISHER

# Mapping of probes to gene symbols
genes.133A <- unique(probes2genes.133A[, 2])

# matrix (note that using also negative controls would be
# matrix(NA,ncol=6,nrow=16))
matriz.fisher <- matrix(NA, ncol = 6, nrow = 6)
colnames(matriz.fisher) <- c("CRC_Down", "Prostate_Cancer_Down", "Lung_Cancer_Down",
    "CRC_Up", "Prostate_Cancer_Up", "Lung_Cancer_Up")
rownames(matriz.fisher) <- c("Schizo_Up", "Alzheimer_Up", "Parkinson_Up", "Schizo_Down",
    "Alzheimer_Down", "Parkinson_Down")

matriz.fisher["Schizo_Up", "CRC_Up"] <- fisher(core.szo.up, core.crc.up, "133A")$p.value
matriz.fisher["Schizo_Up", "Lung_Cancer_Up"] <- fisher(core.szo.up, core.lung.up,
    "133A")$p.value
matriz.fisher["Schizo_Up", "Prostate_Cancer_Up"] <- fisher(core.szo.up, core.prostate.up,
    "133A")$p.value
matriz.fisher["Schizo_Up", "CRC_Down"] <- fisher(core.szo.up, core.crc.down,
    "133A")$p.value
matriz.fisher["Schizo_Up", "Lung_Cancer_Down"] <- fisher(core.szo.up, core.lung.down,
    "133A")$p.value
matriz.fisher["Schizo_Up", "Prostate_Cancer_Down"] <- fisher(core.szo.up, core.prostate.down,
    "133A")$p.value

matriz.fisher["Schizo_Down", "CRC_Up"] <- fisher(core.szo.down, core.crc.up,
    "133A")$p.value
matriz.fisher["Schizo_Down", "Lung_Cancer_Up"] <- fisher(core.szo.down, core.lung.up,
    "133A")$p.value
matriz.fisher["Schizo_Down", "Prostate_Cancer_Up"] <- fisher(core.szo.down,
    core.prostate.up, "133A")$p.value
matriz.fisher["Schizo_Down", "CRC_Down"] <- fisher(core.szo.down, core.crc.down,
    "133A")$p.value
matriz.fisher["Schizo_Down", "Lung_Cancer_Down"] <- fisher(core.szo.down, core.lung.down,
    "133A")$p.value
matriz.fisher["Schizo_Down", "Prostate_Cancer_Down"] <- fisher(core.szo.down,
    core.prostate.down, "133A")$p.value

matriz.fisher["Alzheimer_Up", "CRC_Up"] <- fisher(core.alzh.up, core.crc.up,
    "133A")$p.value
matriz.fisher["Alzheimer_Up", "Lung_Cancer_Up"] <- fisher(core.alzh.up, core.lung.up,
    "133A")$p.value
matriz.fisher["Alzheimer_Up", "Prostate_Cancer_Up"] <- fisher(core.alzh.up,
    core.prostate.up, "133A")$p.value
matriz.fisher["Alzheimer_Up", "CRC_Down"] <- fisher(core.alzh.up, core.crc.down,
```

```r
    "133A")$p.value
matriz.fisher["Alzheimer_Up", "Lung_Cancer_Down"] <- fisher(core.alzh.up, core.lung.down,
    "133A")$p.value
matriz.fisher["Alzheimer_Up", "Prostate_Cancer_Down"] <- fisher(core.alzh.up,
    core.prostate.down, "133A")$p.value

matriz.fisher["Alzheimer_Down", "CRC_Up"] <- fisher(core.alzh.down, core.crc.up,
    "133A")$p.value
matriz.fisher["Alzheimer_Down", "Lung_Cancer_Up"] <- fisher(core.alzh.down,
    core.lung.up, "133A")$p.value
matriz.fisher["Alzheimer_Down", "Prostate_Cancer_Up"] <- fisher(core.alzh.down,
    core.prostate.up, "133A")$p.value
matriz.fisher["Alzheimer_Down", "CRC_Down"] <- fisher(core.alzh.down, core.crc.down,
    "133A")$p.value
matriz.fisher["Alzheimer_Down", "Lung_Cancer_Down"] <- fisher(core.alzh.down,
    core.lung.down, "133A")$p.value
matriz.fisher["Alzheimer_Down", "Prostate_Cancer_Down"] <- fisher(core.alzh.down,
    core.prostate.down, "133A")$p.value

matriz.fisher["Parkinson_Up", "CRC_Up"] <- fisher(core.park.up, core.crc.up,
    "133A")$p.value
matriz.fisher["Parkinson_Up", "Lung_Cancer_Up"] <- fisher(core.park.up, core.lung.up,
    "133A")$p.value
matriz.fisher["Parkinson_Up", "Prostate_Cancer_Up"] <- fisher(core.park.up,
    core.prostate.up, "133A")$p.value
matriz.fisher["Parkinson_Up", "CRC_Down"] <- fisher(core.park.up, core.crc.down,
    "133A")$p.value
matriz.fisher["Parkinson_Up", "Lung_Cancer_Down"] <- fisher(core.park.up, core.lung.down,
    "133A")$p.value
matriz.fisher["Parkinson_Up", "Prostate_Cancer_Down"] <- fisher(core.park.up,
    core.prostate.down, "133A")$p.value

matriz.fisher["Parkinson_Down", "CRC_Up"] <- fisher(core.park.down, core.crc.up,
    "133A")$p.value
matriz.fisher["Parkinson_Down", "Lung_Cancer_Up"] <- fisher(core.park.down,
    core.lung.up, "133A")$p.value
matriz.fisher["Parkinson_Down", "Prostate_Cancer_Up"] <- fisher(core.park.down,
    core.prostate.up, "133A")$p.value
matriz.fisher["Parkinson_Down", "CRC_Down"] <- fisher(core.park.down, core.crc.down,
    "133A")$p.value
matriz.fisher["Parkinson_Down", "Lung_Cancer_Down"] <- fisher(core.park.down,
    core.lung.down, "133A")$p.value
matriz.fisher["Parkinson_Down", "Prostate_Cancer_Down"] <- fisher(core.park.down,
    core.prostate.down, "133A")$p.value

matriz.fisher
```

```
##                 CRC_Down Prostate_Cancer_Down Lung_Cancer_Down     CRC_Up
## Schizo_Up      2.493e-10            5.636e-06        1.857e-26  7.551e-01
## Alzheimer_Up   1.930e-46            3.452e-35        2.914e-76  1.000e+00
## Parkinson_Up   3.129e-04            5.464e-12        4.638e-41  7.109e-12
## Schizo_Down    5.458e-01            9.929e-01        4.256e-01  4.162e-33
## Alzheimer_Down 1.000e+00            1.000e+00        5.803e-01 2.041e-143
## Parkinson_Down 6.451e-02            9.982e-01        3.783e-02  2.058e-24
```

```
##                  Prostate_Cancer_Up Lung_Cancer_Up
## Schizo_Up                 4.286e-01      1.000e+00
## Alzheimer_Up              1.000e+00      1.000e+00
## Parkinson_Up              9.560e-01      1.000e+00
## Schizo_Down               6.500e-20      3.140e-23
## Alzheimer_Down            1.136e-83      1.821e-79
## Parkinson_Down            4.969e-48      3.193e-23

# Write the final fisher result
write.table(matriz.fisher, file = paste(dir.out, "/matrix_results_fisher_",
    "FEM", ".csv", sep = ""), quote = F)

# Multiple testing: pvalues corrected by Bonferroni
matriz.fisher2 <- matriz.fisher * dim(matriz.fisher)[1] * dim(matriz.fisher)[2]
matriz.fisher2[matriz.fisher2 > 1] <- 1

matriz.fisher2

##                 CRC_Down Prostate_Cancer_Down Lung_Cancer_Down     CRC_Up
## Schizo_Up      8.973e-09            2.029e-04        6.686e-25  1.000e+00
## Alzheimer_Up   6.949e-45            1.243e-33        1.049e-74  1.000e+00
## Parkinson_Up   1.126e-02            1.967e-10        1.670e-39  2.559e-10
## Schizo_Down    1.000e+00            1.000e+00        1.000e+00  1.498e-31
## Alzheimer_Down 1.000e+00            1.000e+00        1.000e+00 7.346e-142
## Parkinson_Down 1.000e+00            1.000e+00        1.000e+00  7.409e-23
##                  Prostate_Cancer_Up Lung_Cancer_Up
## Schizo_Up                 1.000e+00      1.000e+00
## Alzheimer_Up              1.000e+00      1.000e+00
## Parkinson_Up              1.000e+00      1.000e+00
## Schizo_Down               2.340e-18      1.130e-21
## Alzheimer_Down            4.089e-82      6.557e-78
## Parkinson_Down            1.789e-46      1.149e-21

# Write the final fisher result corrected by Bonferroni
write.table(matriz.fisher2, file = paste(dir.out, "/matrix_resuls_fisher_",
    "FEM", "_Bonferroni.csv", sep = ""), quote = F)
```

A. Internal Functions

In Part2 and Part3 we make use of functions Part2/functions_part2.R and Part3/functions_part3.R. Here is the code used in Part2/functions_part2.R:

```
# install.packages('MetaDE') install.packages('MetaQC')
# install.packages('MetaPath')

# library(MetaDE) library(MetaQC) library(MetaPath)


# leer.datos function: read normalized data from files previously obtained
# (using fRMA) INPUT: Directory where the normalized data is OUTPUT:
# Normalized data

leer.datos <- function(direct, formato = "txt", skip = 2, log2 = T) {

    ## Format: 1 row with {CASE, CONTROL}; another binary row {1,0} (={case,
    ## control}) y the rest correspond to intensity values (already normalized)
    ## for each probe/gene. Each column is a sample

    ## SYMBOL case1 case2 case3 case4 case5 control1 control2 control3 control4
    ## control5 label NA 1 1 1 1 1 0 0 0 0 0 s01 TP53 7.691989 8.981657 5.719659
    ## 6.124408 6.370273 7.089947 1.700494 8.656502 3.359879 6.057615 s02 TP53
    ## 2.955228 8.035940 5.320929 6.818047 8.275088 5.093363 2.846171 4.817742
    ## 8.008358 4.123111 s03 TP53 7.358284 5.147381 5.506889 4.992996 4.193587
    ## 3.168506 7.601431 5.091096 8.603137 7.681098 s04 TP53 2.013336 4.566419
    ## 4.818600 5.971021 6.327617 7.706960 7.742386 9.528161 9.688512 10.591540
    ## s05 PAX8 1.682574 4.505451 4.861700 6.239061 6.275045 6.477885 7.386773
    ## 7.505806 7.998875 8.498296 s06 PAX8 3.544599 4.092399 4.176114 4.214087
    ## 4.677679 4.760397 4.889076 4.939919 5.173968 5.256308 s07 PAX8 2.680215
    ## 2.810051 2.975162 3.165446 3.778299 4.103315 4.204476 4.350668 4.629691
    ## 6.099961
    ficheros <- list.files(direct)
    files <- c()
    unextended.files <- strsplit(ficheros, split = "\\.")
    for (i in 1:length(unextended.files)) {
        files[i] <- paste(unextended.files[[i]][1:(length(unextended.files[[i]]) -
            1)], collapse = ".")
    }
    normalizado <- MetaDE.Read(paste(direct, files, sep = "/"), skip = rep(skip,
        length(files)), via = formato, matched = F, log = log2)
    normalizado
}


## generar_directorios function : creation of required directories INPUT:
## Path where the results of the Part2 (Meta-analysis) will be saved.
## OUTPUT: Creations of Part2/MetaDE_output (all the results from the
## meta-analysis part), Part2/Effects&Variances,Part2/individual_filtered,
## Part2/individual_analysis, Part2/individual_reads,
## Part2/individual_matched and Part2/individual_merged.

generar_directorios <- function(dir.aux) {
```

```
    lista.directorios <- c(dir.aux, "Part2/MetaDE_output", paste(dir.aux, c("Effects&Variances",
        "individual_filtered", "individual_analysis", "individual_reads", "individual_matched",
        "individual_merged", "meta"), sep = ""))
    for (d in lista.directorios) {
        if (!(file.exists(d))) {
            dir.create(d)
            warning(paste("No existía el directorio ", d, sep = ""))
        }
    }
}


## preprocesar.lista function : MetaDE preprocessing: for each disease, data
## is matched (MetaDE.match), merged (MetaDE.merge) and filtered
## (MetaDE.filter) from the normalized data INPUT: normalized data (part1),
## located in ./Part2/data/disease.name, where disease.name could be
## 'alzheimer', 'asthma', 'colon', 'distrofiaMuscular', 'lung', 'parkinson',
## 'prostate','schizophrenia', 'hiv', 'pulmonarSarcoidosis' and 'malaria'.
## Each folder contains the normalization outputs from every different study
## for that disease (folder name).  OUTPUT: It generates .Rdata files for
## each disease :'./Part2/intermediate_data/individual_reads' (MetaDE.read),
## './Part2/intermediate_data/individual_filtered' (MetaDE.filter),
## './Part2/intermediate_data/individual_matched' (MetaDE.match) and
## './Part2/intermediate_data/individual_merged' (MetaDE.merge)

preprocesar.lista <- function(directorios, dir.aux, p.rep = "IQR", formato = "txt",
    skip = 2, log2 = T, del.perc = c(0, 0), mvperc = 0) {
    for (i in 1:length(directorios)) {
        d <- directorios[i]
        name.d <- names(directorios)[i]

        ## Data reading
        normalizado <- leer.datos(d, formato = formato, skip = skip, log2 = log2)
        save(normalizado, file = paste(dir.aux, "individual_reads/", name.d,
            ".Rdata", sep = ""))
        ## matching
        matcheado <- MetaDE.match(normalizado, pool.replicate = p.rep)
        save(matcheado, file = paste(dir.aux, "individual_matched", "/matcheado_",
            name.d, ".Rdata", sep = ""))

        ## merging
        mergeado <- MetaDE.merge(matcheado, MVperc = mvperc)
        save(mergeado, file = paste(dir.aux, "individual_merged", "/mergeado_",
            name.d, ".Rdata", sep = ""))

        ## filtering
        filtrado <- MetaDE.filter(mergeado, DelPerc = del.perc)
        save(filtrado, file = paste(dir.aux, "individual_filtered", "/filtrado_",
            name.d, ".Rdata", sep = ""))
    }
}

## cargar.preprocesado function : loads previously generated data (from
```

```r
## MetaDE matching, merging and filtering) INPUT : names of the diseases to
## load data for them; and the directory where the data was saved previously.
## OUTPUT: a list with three sublists (with the filtered, merged and matched
## data respectively); each sublist has also an element for each disease
## indicated in the input

cargar.preprocesado <- function(nombres, dir.aux) {
    o <- list()
    o$normalizados <- list()
    o$matcheados <- list()
    o$filtrados <- list()
    o$mergeados <- list()
    for (n in nombres) {
        load(paste(dir.aux, "individual_reads/", n, ".Rdata", sep = ""))
        o$normalizados[[n]] <- normalizado
        load(paste(dir.aux, "individual_matched/matcheado_", n, ".Rdata", sep = ""))
        o$matcheados[[n]] <- matcheado
        load(paste(dir.aux, "individual_merged/mergeado_", n, ".Rdata", sep = ""))
        o$mergeados[[n]] <- mergeado
        load(paste(dir.aux, "individual_filtered/filtrado_", n, ".Rdata", sep = ""))
        o$filtrados[[n]] <- filtrado
    }
    o
}

## cargar.ves function: Loads data from effect sizes and variances previously
## obtained INPUT: names of the diseases and path to the
## 'Part2/intermediate_data' OUTPUT: list with all the effects sizes and
## variances for each disease indicated in the input

cargar.ves <- function(nombres, dir.aux) {
    o <- list()
    for (n in nombres) {
        load(paste(dir.aux, "Effects&Variances/e&v_", n, ".Rdata", sep = ""))
        o[[n]] <- efectos.y.varianzas
    }
    o
}

## various.effect.sizes function : it obtains the effect sizes and variances
## (using MetaDE 'ind.cal.ES' function), and saves these results in .Rdata
## files INPUT: filtered data and name of diseases to work with OUTPUT: A
## list for which each element contains effect sizes and variances for all
## the studies of a particular disease (each element of the list -> one
## disease effect sizes and variances)

various.effect.sizes <- function(filtrados, name) {
    efectos.y.varianzas <- ind.cal.ES(filtrados[[name]], paired = rep(FALSE,
        length(filtrados[[name]])), nperm = 300)
    save(efectos.y.varianzas, file = paste(dir.aux, "Effects&Variances/e&v_",
        name, ".Rdata", sep = ""))
    efectos.y.varianzas
}
```

```r
## FEM function: computes the Fixed Effects Model, and save results in .Rdata
## files INPUT: the output from the cargar.ves function (effects sizes and
## variances data), in a list (each element of the list has the effect sizes
## and variances for all studies of a particular disease); and the name of
## the disease to make the meta-analysis OUTPUT: The outcome of the FEM
## meta-analysis for a given disease (second parameter, name).

FEM <- function(ves, name) {
    fem.out <- MetaDE.ES(ves[[name]], meta.method = "FEM")
    save(fem.out, file = paste(dir.aux, "meta/", name, "_FEM.Rdata", sep = ""))
    fem.out
}
```

Here is the code used in Part3/functions_part3.R:

```r
## fisher function: computes the Fisher's exact test INPUT: List of disease1
## genes (core1), disease2 genes (core2) and the microarray platform OUTPUT:
## The outcome of the fisher.test function

fisher <- function(core1, core2, plataf = "133A") {
    n.genes <- ifelse(plataf == "133A", 14538, ifelse(plataf == "Plus2", 23945,
        0))
    if (n.genes == 0) {
        warning("Entrada de plataforma errónea")
    }
    if (plataf == "133A") {
        core1 <- intersect(core1, genes.133A)
        core2 <- intersect(core2, genes.133A)
    } else {
        core1 <- intersect(core1, genes.plus)
        core2 <- intersect(core2, genes.plus)
    }
    inters <- length(intersect(core1, core2))
    kk <- matrix(c(inters, length(core1) - inters, length(core2) - inters, n.genes +
        inters - length(core1) - length(core2)), nrow = 2, ncol = 2)
    fisher.test(kk, alternative = "greater")
}


## crear.directorios function: Creates the output and intermediate results
## folders, if they do not exist INPUT: Path that will contain the folders to
## create OUTPUT: creation of the input folders if they do not exist

crear.directorios <- function(V) {
    for (v in V) {
        if (!file.exists(v)) {
            dir.create(v)
            warning(paste("No existía el fichero ", v, sep = ""))
        }
    }
}

## obtener.probes2genes function: Read data to associate probes (from
## different platforms) with gene symbols INPUT: Path to the folder
```

```r
## containing the files mapping probes to genes OUTPUT: list with all the
## mapping probes <--> genes for different platforms

obtener.probes2genes <- function(dir.sondas2genes) {
    S <- scan(paste(dir.sondas2genes, "probes_to_genes_95_biomart.csv", sep = "/"),
        what = character(0), sep = "\n")
    probes2genes.u95 <- matrix(unlist(strsplit(S, "\t")), nrow = length(S),
        ncol = 2, byrow = T)
    S <- scan(paste(dir.sondas2genes, "probes_to_genes_plus_biomart.csv", sep = "/"),
        what = character(0), sep = "\n")
    probes2genes.plus <- matrix(unlist(strsplit(S, "\t")), nrow = length(S),
        ncol = 2, byrow = T)
    S <- scan(paste(dir.sondas2genes, "probes_to_genes_133A_biomart.csv", sep = "/"),
        what = character(0), sep = "\n")
    probes2genes.133A <- matrix(unlist(strsplit(S, "\t")), nrow = length(S),
        ncol = 2, byrow = T)
    S <- scan(paste(dir.sondas2genes, "probes_to_genes_133A2_biomart.csv", sep = "/"),
        what = character(0), sep = "\n")
    probes2genes.133A2 <- matrix(unlist(strsplit(S, "\t")), nrow = length(S),
        ncol = 2, byrow = T)

    z <- list()
    z$u95 <- probes2genes.u95[probes2genes.u95[, 1] != "", ]
    z[["133A"]] <- probes2genes.133A[probes2genes.133A[, 1] != "", ]
    z[["133A2"]] <- probes2genes.133A2[probes2genes.133A2[, 1] != "", ]
    z$plus <- probes2genes.plus[probes2genes.plus[, 1] != "", ]
    z
}

## obtener.listas function: INPUT: Takes the path where the output files from
## Meta-analysis (part2) are and the type of the Meta-analysis OUTPUT: list
## containing sublists for the downregulated (respectively upregulated) genes
## for each disease, obtained in the meta-analysis

obtener.listas <- function(direct = ".", type = "FEM") {

    L <- list()

    L$core.alzh.up <- as.character(unlist(read.table(paste(dir.listas, "/alzheimer_up_",
        type, sep = ""), stringsAsFactors = F)))
    L$core.alzh.down <- as.character(unlist(read.table(paste(dir.listas, "/alzheimer_down_",
        type, sep = ""), stringsAsFactors = F)))
    L$core.park.up <- as.character(unlist(read.table(paste(dir.listas, "/parkinson_up_",
        type, sep = ""), stringsAsFactors = F)))
    L$core.park.down <- as.character(unlist(read.table(paste(dir.listas, "/parkinson_down_",
        type, sep = ""), stringsAsFactors = F)))
    L$core.szo.up <- as.character(unlist(read.table(paste(dir.listas, "/schizophrenia_up_",
        type, sep = ""), stringsAsFactors = F)))
    L$core.szo.down <- as.character(unlist(read.table(paste(dir.listas, "/schizophrenia_down_",
        type, sep = ""), stringsAsFactors = F)))

    L$core.lung.up <- as.character(unlist(read.table(paste(dir.listas, "/lung_up_",
        type, sep = ""), stringsAsFactors = F)))
    L$core.lung.down <- as.character(unlist(read.table(paste(dir.listas, "/lung_down_",
```

```r
        type, sep = ""), stringsAsFactors = F)))
    L$core.prostate.up <- as.character(unlist(read.table(paste(dir.listas, "/prostate_up_",
        type, sep = ""), stringsAsFactors = F)))
    L$core.prostate.down <- as.character(unlist(read.table(paste(dir.listas,
        "/prostate_down_", type, sep = ""), stringsAsFactors = F)))
    L$core.crc.up <- as.character(unlist(read.table(paste(dir.listas, "/colon_up_",
        type, sep = ""), stringsAsFactors = F)))
    L$core.crc.down <- as.character(unlist(read.table(paste(dir.listas, "/colon_down_",
        type, sep = ""), stringsAsFactors = F)))

    L$core.asthma.up <- as.character(unlist(read.table(paste(dir.listas, "/asthma_up_",
        type, sep = ""), stringsAsFactors = F)))
    L$core.asthma.down <- as.character(unlist(read.table(paste(dir.listas, "/asthma_down_",
        type, sep = ""), stringsAsFactors = F)))
    L$core.hiv.up <- as.character(unlist(read.table(paste(dir.listas, "/hiv_up_",
        type, sep = ""), stringsAsFactors = F)))
    L$core.hiv.down <- as.character(unlist(read.table(paste(dir.listas, "/hiv_down_",
        type, sep = ""), stringsAsFactors = F)))
    L$core.malaria.up <- as.character(unlist(read.table(paste(dir.listas, "/malaria_up_",
        type, sep = ""), stringsAsFactors = F)))
    L$core.malaria.down <- as.character(unlist(read.table(paste(dir.listas,
        "/malaria_down_", type, sep = ""), stringsAsFactors = F)))
    L$core.distrofia.up <- as.character(unlist(read.table(paste(dir.listas,
        "/distrofiaMuscular_up_", type, sep = ""), stringsAsFactors = F)))
    L$core.distrofia.down <- as.character(unlist(read.table(paste(dir.listas,
        "/distrofiaMuscular_down_", type, sep = ""), stringsAsFactors = F)))
    L$core.sarcoidosis.up <- as.character(unlist(read.table(paste(dir.listas,
        "/pulmonarSarcoidosis_up_", type, sep = ""), stringsAsFactors = F)))
    L$core.sarcoidosis.down <- as.character(unlist(read.table(paste(dir.listas,
        "/pulmonarSarcoidosis_down_", type, sep = ""), stringsAsFactors = F)))
    L
}
```
